# The effectiveness of mobile app-based interventions in facilitating behaviour change towards healthier and more sustainable diets: a systematic review and meta-analysis

**DOI:** 10.1186/s12966-025-01823-7

**Published:** 2025-09-30

**Authors:** Esther Curtin, Rosemary Green, Kerry A. Brown, Sarah Nájera Espinosa, Abinaya Chandrasekar, Lily Hopkins, Grace Turner, Carmelia Alae-Carew, Karen Ullian, Pauline Scheelbeek

**Affiliations:** 1 Department of Population Health, Faculty of Epidemiology and Population Health, London School of Hygiene & Tropical Medicine, London, WC1E 7HT UK; 2 University of Exeter Medical School, Faculty of Health & Life Sciences, Exeter, EX1 2HZ UK; 3 Department of Non-Communicable Disease Epidemiology, Faculty of Epidemiology and Population Health, London School of Hygiene & Tropical Medicine, London, WC1E 7HT UK; 4 Department of Public Health, Environments and Society, Faculty of Public Health and Policy, London School of Hygiene & Tropical Medicine, London, WC1H 9SH UK

**Keywords:** Sustainable diets, Meat consumption, Healthy eating, Nutrition, Dietary change, Behaviour change, MHealth, Interventions, Systematic review, Meta-analysis

## Abstract

**Background:**

Digital health apps have been shown to influence healthy eating, but whether they can promote environmentally friendly diets remains unclear. In countries where diets typically contain a high proportion of carbon-intensive foods (e.g., meat), transitioning to healthier alternatives (e.g., fruit, vegetables, and legumes) can substantially reduce food-related greenhouse gas emissions while also improving public health. Our systematic review and meta-analysis aimed to investigate the effectiveness of mobile apps in facilitating more sustainable and healthier diets among adults from high-income countries.

**Methods:**

We searched MEDLINE, EMBASE, PsycINFO, CINAHL, Global Health, GreenFILE, Web of Science, Cochrane Trials, and ClinicalTrials.gov from inception until Jan 20, 2025. We included studies that reported the effects of app-based behavioural interventions on the consumption of fruit, vegetables, legumes, nuts, fish, dairy, and/or meat, compared to a control, baseline period, or different intervention. For outcomes measured in at least two studies, we conducted random effects meta-analysis and meta-regression to understand population differences and the role of specific intervention components.

**Results:**

Of the 7356 records screened, 21 were included. Nine studies scored ‘high’, 10 scored ‘fair’, and two scored ‘low’ for reporting transparency. Combined, the studies analysed 12,898 participants, interventions lasted from three days to six months, and outcomes were assessed up to 12 months post-intervention. Forty percent of studies targeted populations with at least one health risk factor and 81% of study populations did not meet dietary guidelines at baseline for their primary outcome. Meta-analyses indicated that app use led to increased fruit and vegetable consumption (0.48 portions/day, 95% CI 0.18, 0.78, *p* = 0.002) and a small decrease in meat consumption (-0.10 portions/day, 95% CI -0.16, -0.03, *p* = 0.004), with meat-focussed apps showing as more effective than general apps for meat reduction. Meta-regression also revealed that message-based content was particularly effective in promoting meat reduction. There were no pronounced effects on legume or dairy consumption, or differences across populations.

**Conclusion:**

Our results indicate that apps could be a valuable addition to the multiple interventions required to promote sustainable diets. However, to strengthen the evidence for outcomes beyond fruit and vegetables, we need standardised reporting of populations and intervention components.

**Supplementary Information:**

The online version contains supplementary material available at 10.1186/s12966-025-01823-7.

## Background

The global food system accounts for one third of greenhouse gas emissions [1], primarily driven by the production, processing, and consumption of animal-sourced foods [2]. Poor diets are also a leading cause of obesity [3], which is a serious public health challenge with substantial economic and social costs [4]. Diets in high-income countries (HICs) typically contain low amounts of fruit, vegetables, and legumes, which are rich in fibre and essential micronutrients [5]. Conversely, consumption of animal protein and foods high in fat, salt, and sugar often exceeds recommended levels [5]. HICs therefore play a critical role in realising reductions in diet-related emissions [6]. In contrast, the situation is more complex in low- and middle-income countries (LMICs) where increasing access to animal-sourced foods for some could help address nutritional deficiencies [7]. While the intake of red and processed meat has declined in some high greenhouse gas emitting countries (e.g., from 74 to 56g/day in the period 2008 to 2019 in the UK) [8], average meat intake has increased across all HICs in recent years (from 240 to 252g/day in the period 2011 to 2021) [9]. Interventions are urgently needed that effectively change food demand and consumption, thereby increasing the sustainability of the food system and improving health [10]. Several studies have assessed the long-term health benefits of vegetarian and vegan diets (e.g., vegetarians had a 23% lower heart disease risk than omnivores among 65,000 UK residents) [11]. However, there are concerns about the achievability and longevity of a population-level shift towards meat-free diets. Thus, flexitarian, plant-forward diets that emphasise plant-based foods but are not completely devoid of meat (such as the EAT-Lancet diet), may be more feasible [12].

Smartphone applications (apps) as tools to encourage dietary change can function as information/education providers, diet/weight trackers, barcode scanners, recipe builders, or a combination of these [13]. Previous systematic reviews have documented the effectiveness of app use in facilitating dietary improvement [14–16], but whether apps can promote environmentally friendly diets remains unclear. In light of this, elucidating the effects on foods with high carbon footprints (e.g., meat) as well as their healthy substitutes (e.g., fruit, vegetables, and legumes) provides a good starting point to understanding the role of apps in sustainable dietary transitions. There is also a lack of evidence on which features of apps are most effective for influencing consumption across these key food groups, and how best to deliver them, with previous literature focusing on the behaviour change techniques (BCTs) employed [14–16], rather than how they are implemented. Moreover, since levels of app usage, engagement, and effectiveness have been associated with social and demographic factors [17, 18], regulatory bodies recommend using apps as a supplement to face-to-face interventions to enhance reach of timely and personalised dietary advice [19]. However, these public health recommendations are largely based on evidence from clinical settings and the effects of interventions delivered solely via mobile apps for the general public remain under-researched [20].

Given no previous systematic reviews and meta-analyses exist on this topic, the aim of this systematic review and meta-analysis was to synthesise existing literature on the effectiveness of mobile app-based interventions in facilitating behaviour change towards more sustainable and healthier diets, in terms of the consumption of fruit, vegetables, legumes, nuts, fish, dairy, and/or meat, among non-clinical adult populations residing in HICs. We also assessed behavioural changes across population groups, defined by the studies’ target characteristics and whether they met food-based dietary guidelines, and the effects of individual app features, defined by the BCTs and delivery techniques employed.

## Methods

This systematic review was pre-registered on PROSPERO (ID: CRD42023374287) [21], and reporting followed the Preferred Reporting Items for Systematic reviews and Meta-Analyses (PRISMA) [22].

### Search strategy and selection criteria

We searched seven databases (MEDLINE, EMBASE, GreenFile, CINAHL, PsycINFO, Global Health, and Web of Science) and two trial registries (Clinicaltrials.gov and Cochrane Trials) for articles up to Jan 20, 2025, with no restrictions on publication year but limited to articles originally published in, or officially translated to, English. No translation tools were employed due to the lack of funding available for this review. We included two sets of search terms based on sustainable and healthy diets and mobile apps and refined them with the input of an information specialist (see Supplementary Table 1, Additional File 1). Relevant protocols from the database search were reviewed for any recently published results. We also applied a simplified search string to the advanced Google Chrome search engine, and, using the SEOquake plug-in, exported the first 600 URLs and screened them in MS Excel. Lastly, we sought expert recommendations to identify possible missing articles.

After de-duplication in Rayyan [23], titles and abstracts were independently screened according to the eligibility criteria (see Supplementary Table 2, Additional File 1); EC screened all records and LH, AC, GT, CA, and KU each screened a proportion. The screening of the full-text records was also performed in duplicate. We recorded reasons for exclusion and discussed conflicts. Studies were included if they a) evaluated a mobile app-based dietary intervention, b) recruited adults, c) were conducted in a HIC as per the World Bank classification (see Supplementary Table 3, Additional File 1) [24], and d) reported intervention effects on the consumption of one of six food groups important for sustainability and health (fruit and/or vegetables, legumes, nuts, fish, dairy, and meat). We included randomised controlled trials (RCTs), non-randomised controlled trials, and studies with pre-post designs, as both randomised and non-randomised studies can be employed to address questions around effectiveness in real world settings [25]. Interventions could be single- or multi-component provided the intervention arm used an app in a manner that was distinguishable from the control arm.

Studies were excluded if they a) did not provide the full text in English, b) evaluated an app-based intervention delivered in a clinical setting by a trained dietician, for example, c) assessed proxies for consumption, e.g., intention to consume specific foods or biomarkers associated with consumption of certain nutrients, or d) recruited participants with dietary restrictions or diet-related conditions, e.g., diabetes or hypertension. Eight public advisors participated in a workshop where they shared their experience of using apps while managing specific health conditions. This discussion led to a consensus to exclude studies of populations with any dietary restrictions or requirements like pregnant women or those recovering from disease in view of increasing comparability.

### Data extraction

EC extracted all data, while LH extracted data from a random 10% of studies. Any discrepancies were resolved through discussion. Missing information was obtained from study protocols or by contacting authors.

Data extracted on the study populations included sociodemographic characteristics and baseline food consumption. The study populations were categorised in two ways.

1. Targeted/general: a study sample was labelled as “targeted” if the participants were recruited based on known risk factors for poor diets, such as living with obesity or being part of a minority ethnic group [26]. Otherwise, they were labelled as “general”.
2. Not meeting/meeting dietary guidelines: a study sample was labelled as “not meeting” if the average baseline dietary intake fell short of the recommended intake defined by the respective national guidelines for the primary food group(s), and as “meeting” if it met the recommendation (see Supplementary Tables 4 and 5, Additional File 1).

Data extracted on the interventions included the BCTs and delivery techniques featured in the apps. Example components are defined in Table 1, and all components are defined in Supplementary Table 6, Additional File 1. EC and LH both completed the online BCT Taxonomy training, with the aim of improving consistency in coding. Interventions were also categorised according to the specificity of their behavioural focus. These variables are described below.

1. BCTs, the active components in the intervention designed to facilitate the desired behavioural change, were identified using the BCT Taxonomy Version 1 (BCTTv1) [27]. This taxonomy includes 93 BCTs organised into 16 clusters.
2. Delivery techniques, which determine how BCTs are operationalised in practice to increase their functionality and user-friendliness, were selected from the Behavioural Intervention Technology (BIT) model [28] and Mode of Delivery Ontology (MoDO) [29].
3. Specific/general: an intervention was labelled as “specific” if it exclusively focused on influencing the measured food group(s), and “general” if the intervention focused on food groups other than those measured.

**Table 1:** Brief definitions of a sample of intervention components within the included apps

**Behaviour Change Techniques**	
Goals and planning	Providing practical tools to enable creating goals for behaviours or outcomes of behaviours and making progress towards them
Natural consequences	Providing information on the health, social, environmental, and emotional outcomes of a behaviour
**Delivery Techniques**	
Information delivery	Typically involves one-way interactions in which the app provides content to the user. This can include text, video, images, audio, or a combination of media
Messaging	Forms include one-way or bidirectional interactions with the intervention lead, discussion boards with other participants as a social support tool, or notifications to aid compliance (e.g., food log reminders)
Media	Refers to content delivered in text, video, image, or audio format. The medium can differ for various elements of the intervention to make them more engaging and/or informative for the user
Personalisation	Tailoring the intervention using user-determined criteria, e.g., behavioural goals, to adapt the intervention content based on the user’s past behaviour and/or context. Examples of personalised elements included action plans and feedback reports

### Study transparency assessment

We created our own checklist covering reporting transparency and potential bias arising from the studies (see Supplementary Table 7, Additional File 1). This enabled a score that was comparable across studies regardless of design. We built on established checklists and adapted items from the Consolidated Standards of Reporting Trials (CONSORT) [30], Risk of Bias 2 (RoB2) [31], and Template for Intervention Description and Replication (TIDieR) [32]. Potential bias was identified under any of the following conditions:

- Attrition: fewer than 60% of participants remained in the study at follow-up.
- Selection: a statistically significant difference in demographic characteristics and/or baseline intake between those who dropped out and the original sample.
- Reporting: dietary intake was measured using a single question rather than validated tools.
- Funding: the study received financial support from industry sources.

Criteria were scored as 1 (fulfilled), 0.5 (partially fulfilled), or 0 (not fulfilled or unclear). Scores ranged from 0–26 for RCTs and 0–23 for non-RCTs, given the additional three items relating to blinding for RCTs. Higher scores indicated higher reporting quality for the studies. Percentage of fulfilled criteria was obtained by dividing scores by the maximum score and multiplying by 100, categorised into ‘high’ (≥ 66.6%), ‘fair’ (50%−66.6%), and ‘low’ (< 50%) [15].

### Data analysis

Data cleaning and descriptive analyses were conducted using MS Excel (version 16.93.1). R (version 4.3.1) was used for bivariate analysis, meta-analysis, and meta-regression using the packages meta, metafor, dplyr, and forestploter.

For consistency, we converted all outcome data into portions per day. When data were reported in grams, we used the portion sizes set out by at least two regulatory bodies from HICs where the included studies were set to calculate portions per day (see Supplementary Table 8, Additional File 1). The portion size for legumes was defined as the quantity considered a protein source rather than a portion of vegetables.

To look at the results overall, we presented the effects graphically, with the follow-up times (weeks from baseline) on the x axis and effect sizes (change in portions/day) on the y axis, separated by food category.

We computed effectiveness ratios to explore which intervention components (BCTs and delivery techniques) showed promise in influencing the dietary outcomes [33]. We first counted the frequency of interventions that contained each BCT and delivery technique separately and the frequency of “effective” interventions (those that were reported to facilitate a significant change in one of the study’s primary outcomes at the longest follow-up) that contained each component. We then calculated effectiveness ratios (E) using the equation below, and those with ratios > 50%, from at least five interventions, were considered potentially effective.

$$E=\frac{\text{f}{ (effective)}_{i}}{\text{f}{ }_{i}}*100$$

where f is the frequency of a specific component (i) used in the included studies. We also counted the frequency of combinations of BCTs and delivery techniques, i.e., which BCTs were operationalised through which delivery techniques.

Behaviour change outcomes assessed in two or more studies were synthesised using random effects meta-analysis with heterogeneity assessed by Q-statistics. Forest plots were created with the size of the box representing the weight in the analysis. Funnel plots and the Egger’s test assessed publication bias. We included a single effect size per study to deal with multiplicity [34]. For studies reporting multiple time-points, the effect from the longest follow-up was taken. For studies reporting multiple measurement methods, the effect from the most validated measure was taken. For studies reporting multiple analysis approaches, the effect from the intention-to-treat analysis was taken over per-protocol. For studies reporting on fruit and/or vegetables, we took the combined effect if available, and the vegetable effect if fruit and vegetables were reported separately. For studies reporting on meat, we took the total meat effect if available, and the processed meat effect if red and processed meat were reported separately. To increase power in the analysis, within- and between-group comparisons were synthesised together. To avoid duplicating samples from studies with multiple arms, we reported within-group results for two of the RCTs with more than one intervention arm.

To enhance comparability to previous studies and dietary guidelines, effect sizes were unstandardised and the main meta-analysis summarised the effects of studies classified as evaluating “general” interventions. A sensitivity analysis was conducted to compare overall effect sizes from the meta-analyses between “specific” and “general” interventions, with differences assessed by the chi-squared test and level of significance.

We performed meta-regression to explore the predictivity of BCTs, delivery techniques, and characteristics of study participants on the magnitude of change in each food group. One regression model was developed for each food group (i.e., a fruit and vegetable model, a legume model, and a meat model). A dairy model could not be developed due to limited data points. Exposure variables used to populate each meta-regression model were based on bivariate analysis (t-tests and Pearson’s r correlations) for a selected group of BCTs and delivery techniques that showed promise in the effectiveness ratio analysis. Potential confounders related to participant characteristics and study design were identified in the literature [15, 35]. If, in the bivariate analysis, a variable was associated with dietary change at the 10% significance level, the variable was included in the meta-regression. Variance inflation factors (VIF) were calculated for each explanatory variable to check for multicollinearity [36].

## Results

**Fig. 1:**
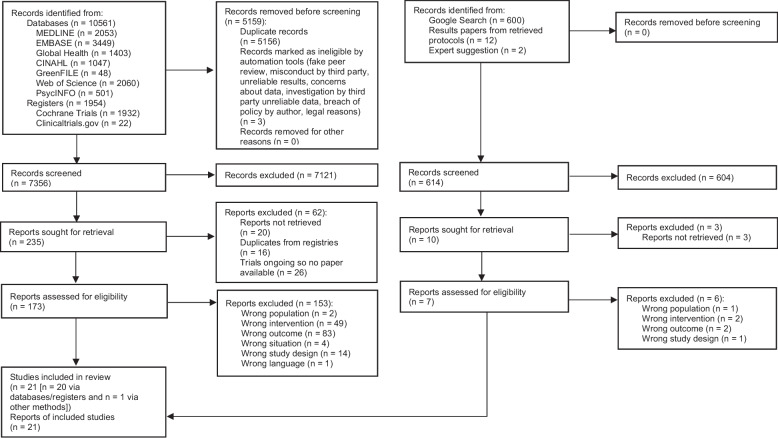
PRISMA flow diagram

The database and registry search yielded 12,515 records. Following de-duplication, 7,356 titles and abstracts were screened. From the 235 eligible for full-text screening, 20 could not be retrieved, through institutional access or by searching the British Library, and/or after contacting information specialists or the authors, 16 records were secondary results papers, and 26 were ongoing studies so were excluded. Of the 173 records screened at full text, 20 were included, and 153 were excluded with reasons (listed in Supplementary Table 9, Additional File 1). Of the 614 records identified via other methods, seven were eligible for full-text screening, and one was included. In total, 21 studies were included in the review (described in Fig. 1) [37–57].

### Study characteristics

Of the 21 studies, 15 studies were RCTs, one was a non-randomised controlled trial, and five were pre-post trials (see Supplementary Table 10, Additional File 1). The total recruited sample across studies was 78,469 with 12,898 (16.4%) remaining at follow-up. The median retention rate was 79%, but the overall rate was driven down by one study with a particularly large baseline sample size (*n* = 68,942) and loss to follow-up (*n* = 8305 at follow-up) [54]. With this study excluded, the overall retention rate was 48.2% and the median retention rate was 85.8%.

Two studies included multiple intervention arms, resulting in a total of 25 interventions evaluated. The studies included multiple outcomes and follow-up assessments, leading to 81 effect sizes across six dietary outcomes: fruit and vegetables (*n* = 38), meat (*n* = 20), legumes (*n* = 14), dairy (*n* = 5), fish (*n* = 2), and nuts (*n* = 2) (see Supplementary Table 11, Additional File 1).

Eight studies were conducted in the USA, five in the UK, two in Spain, and one study each was conducted in Italy, China, the Netherlands, Australia, New Zealand, Switzerland, and Puerto Rico. The proportion of female participants ranged from 0–100% (median = 71.8%). The mean age of recruited populations ranged from 19–59 years (median = 40.7 years), with one study not reporting age. Eight of the studies (40%) targeted participants with at least one health risk factor. The majority (81%) of study populations did not meet the dietary recommendations for their outcome of interest at baseline (see Supplementary Table 5, Additional File 1). The four study populations that met the recommendations were based on portions of fruit and vegetables. Socioeconomic characteristics were poorly and inconsistently reported in some studies, with only 12 studies reporting on education level and eight studies reporting on ethnicity.

**Fig. 2:**
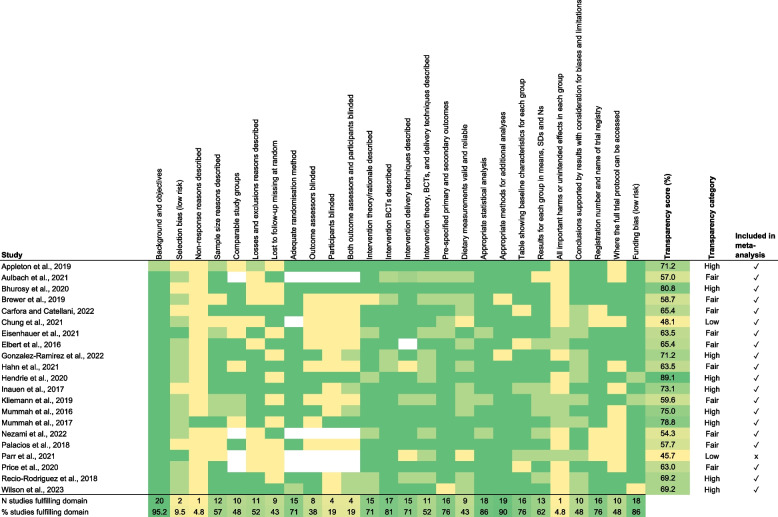
Transparency assessment for the included studies

The average transparency score was 65.7%, with nine studies (42.9%) classified as high, 10 (47.6%) as fair, and two (9.5%) as low reporting transparency (Fig. 2). The low scoring studies used non-randomised controlled and pre-post designs. One had high attrition, and the other did not report the sample size at follow-up. Overall, the most frequently missed details included attempts to limit selection bias (10%), reasons for non-response (5%), and unintended outcomes/harms (5%).

### Intervention characteristics

The evaluated interventions lasted between two days and six months (median = 12 weeks), while the follow-up periods ranged from two days to 12 months (median = 12 weeks). Most studies were focused on general healthy eating and/or fruit and vegetable consumption. Only one app was specifically framed around sustainable diets, delivering four interventions [41]. Most apps were either researcher-developed (*n* = 11, 44%) or co-developed by researchers, practitioners, and industry (*n* = 6, 24%). Researcher-developed apps were most frequently evaluated through RCTs and targeting a single food group, whereas the co-developed apps were more often evaluated through pre-post trials and had a broader scope.

**Fig. 3:**
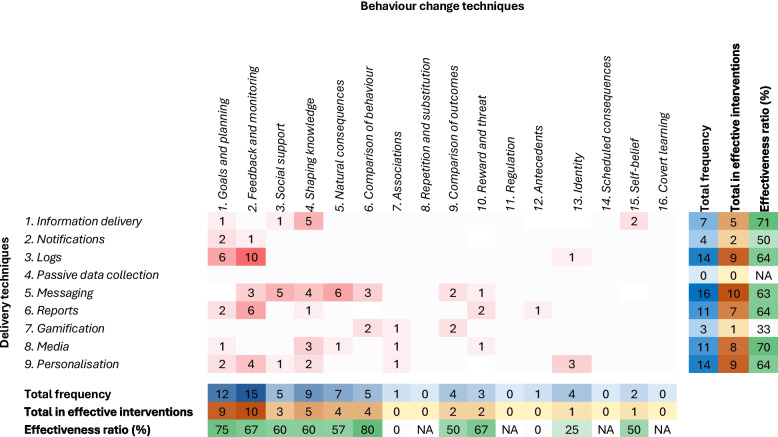
Heat map showing the frequency of intervention components and their effectiveness ratios. The colours represent the frequency; the darker red represents higher frequency of the BCT*delivery technique combination, the darker blue represents higher frequency of each component overall, the darker orange represents higher frequency in the effective interventions, and the darker green represents higher effectiveness ratios

Shown in Fig. 3, the most common BCTs were under the clusters “Feedback and monitoring” and “Goals and planning”, and the most common delivery techniques were “Messaging” and “Personalisation”. In total, 12 BCT clusters and eight delivery techniques were represented. The application of delivery techniques varied across the BCTs, with “Messaging” used in nine clusters, followed by “Personalisation” in six, while “Media” and “Reports” were applied to five clusters. Certain delivery techniques were differentially applied across BCTs, for instance, “Reports”, “Logs”, and “Personalisation” were used most often for “Feedback and Monitoring”, while “Media” and “Messaging” were applied more broadly across clusters. A BCT cluster may have been operationalised through multiple delivery techniques in one intervention, which explains how the total combinations exceed the number of measured interventions for “Feedback and monitoring”. The combinations are described in more detail in Supplementary Table 12, Additional File 1. Most of the studies (*n* = 6, 75%) that targeted specific populations included personalised elements in their intervention.

### Descriptive analyses

The effectiveness ratios in Fig. 3 were calculated considering the intervention components present in the 15 studies reporting a significant change in one of their primary variables (see Supplementary Table 11, Additional File 1). “Goals and planning”, “Feedback and monitoring”, “Social support”, “Shaping knowledge”, Natural consequences”, and “Comparison of behaviour” (BCTs) and “Information delivery”, “Logs”, “Messaging”, “Reports”, “Media”, and “Personalisation” (delivery techniques) were deemed potentially effective (ratios > 50% and present in at least five interventions).

Most interventions facilitated dietary change in the intended direction, with fruit, vegetable, and legume consumption increasing, and meat decreasing post-intervention (see Supplementary Fig. 1, Additional File 2). For nuts, dairy, and fish, the effects were clustered around the x axis, indicating limited evidence of change. The distribution of data points suggested that behaviour change was generally more pronounced around 4–12 weeks post-enrolment, with effects diminishing over time.

### Meta-analyses

**Fig. 4:**
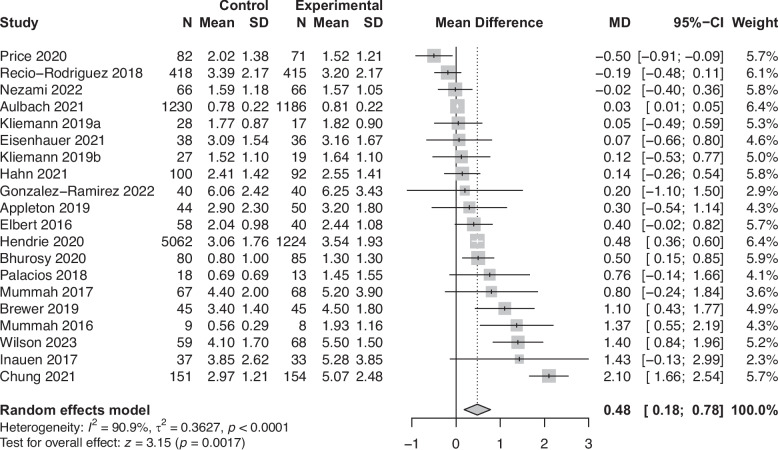
Forest plot of the app intervention effect on fruit and vegetable consumption

**Fig. 5:**
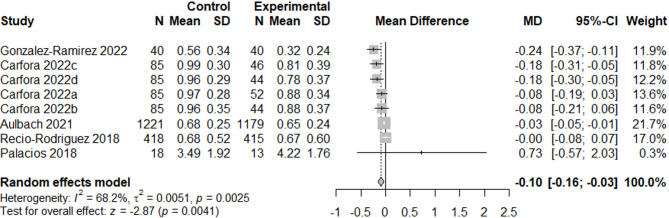
Forest plot of the app intervention effect on meat consumption

**Fig. 6:**
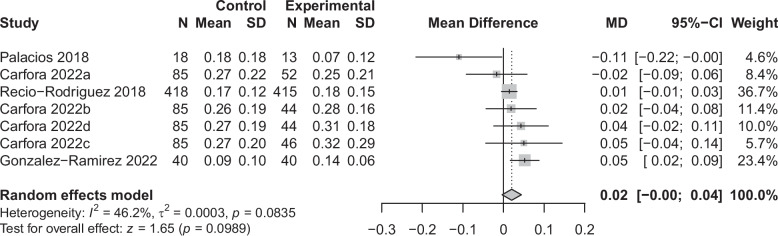
Forest plot of the app intervention effect on legume consumption

**Fig. 7:**
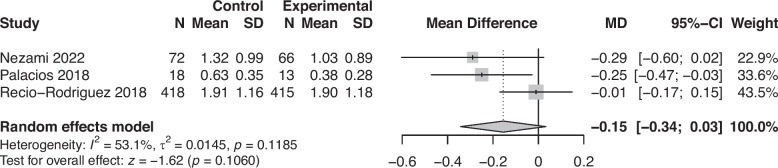
Forest plot of the app intervention effect on dairy consumption

We excluded the particularly large study [54], which was classified as having dubious quality, from all following analyses so that the large sample size did not skew the results. Therefore, a total of 38 results were included in the meta-analyses (*n* = 20 for fruit and vegetables, *n* = 7 for legumes, *n* = 3 for dairy, and *n* = 8 for meat), which included 19 between-group results and 19 within-group results. The meta-analyses showed a positive effect of app use on increasing fruit and vegetable consumption (0.48 portions/day, *p* = 0.002, *n* = 11389, see Fig. 4) and a small effect on decreasing meat consumption (−0.10 portions/day, *p* = 0.004, *n* = 3870, see Fig. 5). We also found small non-significant effects on increasing legume consumption (0.02 portions/day, *p* = 0.10, *n* = 1470, see Fig. 6) and decreasing dairy consumption (−0.15 portions/day, *p* = 0.10, *n* = 1002, see Fig. 7). The Q-statistic for heterogeneity among study results was significant for fruit and vegetables (Q(19) = 208.2, *p* < 0.0001) and meat (Q(7) = 22.0, *p* = 0.002), suggesting considerable variation beyond chance. For legumes (Q(6) = 11.2, *p* = 0.08) and dairy (Q(2) = 4.27, *p* = 0.12), there was less evidence of heterogeneity. Funnel plots did not reveal pronounced asymmetry for any of the outcomes (see Supplementary Fig. 2, Additional File 2), but the Egger’s regression indicated possible evidence of publication bias in smaller trials for fruit and vegetables (*p* = 0.02). The Egger’s regression results for legumes (*p* = 0.69), dairy (*p* = 0.29), and meat (*p* = 0.17) indicated less bias. The test for subgroup differences (Supplementary Table 13, Additional File 1) suggested a statistically significant subgroup effect (*p* = 0.0002) for meat consumption, meaning that the meat effect favoured the more specific apps over the general apps. There were no subgroup differences between the two categories of apps for fruit and vegetables, legume, and dairy consumption.

The bivariate analyses showed that the presence of the BCT “Natural consequences” was linked with increased effectiveness across all outcomes, while the remaining variables differed across outcomes (see Supplementary Table 14, Additional File 1). The meta-regression model building on the bivariate results for fruit and vegetable consumption included two variables (Natural consequences and Comparison of behaviour). Neither of these variables significantly influenced the change in fruit and vegetable consumption (see Supplementary Table 15, Additional File 1). The meta-regression for legumes considered eight explanatory variables (Analysis type, Dietary measurement, Sex, Transparency score, Natural consequences, Messaging, Reports, and Personalisation). VIF values were high in the first model, so Dietary measurement and Sex were removed to avoid overfitting. In the final model, Transparency score approached a significant positive association with legume consumption (+ 0.01 additional portions, *p* = 0.06), suggesting higher quality studies reported slightly larger changes in legume intake. The meta-regression model for meat included nine explanatory variables (Analysis type, Dietary measurement, Sex, Transparency score, Baseline dietary intake, Goals and planning, Natural consequences, Messaging, and Personalisation). VIF values were high for the first model, so Dietary measure, Sex, Transparency, and Baseline intake were removed. The final model indicated that the number of meat portions reduced by an additional 0.24 portions (*p* = 0.03) when apps included messaging components. However, these results should be interpreted with caution considering possible residual multicollinearity indicated by the VIF values.

## Discussion

### Effectiveness of apps in facilitating dietary change

This systematic review evaluated the effect of mobile app-based interventions on promoting sustainable and healthy dietary changes among adults in HICs. Our findings showed that apps generally led to favourable changes in food consumption, peaking around 12 weeks after initiating app use. Overall, we found strong evidence of app-induced increases in fruit and vegetable consumption, with a smaller significant effect observed for decreased meat consumption. Changes in legume consumption (increase) and dairy consumption (decrease) were observed but were non-significant. Additional analyses suggested that, across all outcomes, providing information on the causes and consequences of dietary change showed promise in influencing behaviour. We found evidence that meat-focussed apps, compared to general ones, and message-based components were particularly effective in encouraging meat reduction. We found no pronounced differences in the effects of apps on behaviour change according to population characteristics.

Our results align with previous reviews of app-based interventions documenting dietary improvements [14, 15]. Notably, a previous meta-analysis of social media interventions reported an increase in fruit and vegetable intake of 0.35 portions per week [16], a smaller effect size than ours. As we looked at standalone mobile apps, our results convey that more specific, targeted apps may facilitate larger behavioural changes than generic ones. Our findings around the effects of individual intervention components extend, and somewhat differ to, previous work. We found message-based content to be particularly effective, which is consistent with established health communication research showing the effects of targeted text messages and reminders [58]. Meanwhile, a past systematic review found no added benefit of individual BCTs [15], though their analysis combined dietary behaviour and anthropometric outcomes, potentially diluting the effects. In addition, unlike previous systematic reviews that have reported benefits of remote monitoring and feedback on dietary behaviour [59, 60], we found no substantial benefits of these features for individual food groups. This could be because these autonomy-supporting strategies tend to be more effective for changing consumption of foods high in fat, salt, and/or sugar, which participants may be extrinsically motivated to engage in when recruited from healthcare settings. Self-monitoring may also work better when paper-based tracking tools accompany digital methods [59] and human-generated feedback may be favourable over algorithm-based feedback [60], which our review did not explore. Moreover, a notable evidence gap was the lack of studies incorporating “Passive data collection”, a common feature in supermarket apps that collect data in an opt-out fashion, for example. Addressing this gap in future research may help to monitor and optimise the benefits of app-based interventions on dietary behaviour at a population level, without requiring participants to track their own progress.

### Implications for policy and practice

Global estimates indicate that adults in HICs consume on average three portions of fruit and vegetables, one fifth of a portion of legumes, over two portions of dairy, and one portion of meat per day [61]. Given these baseline consumption patterns, our results suggest that if the effects we found were extrapolated over a population, mobile apps could help bring diets closer to the recommended intake of fruit and vegetables, whereas multi-component strategies beyond apps may be required to meet the recommendations for legumes, dairy, and meat [7].

Since most study populations did not meet their respective national dietary guidelines at baseline, our findings are likely to be generalisable to real-world settings. In the UK for example, less than 0.1% of adults adhere to all dietary guidelines [62]. Our results therefore suggest that the nutrition-focused health apps evaluated in our sample could be relevant for a wide range of individuals and, unlike physical activity apps – often disproportionately used by already healthy, active people – may not exacerbate existing health equalities [17]. While insufficient data prevented us from comparing outcomes across socioeconomic subgroups, it is well established that people of lower socioeconomic status experience poorer health outcomes and higher mortality rates [17, 63]. Importantly, we found no differences in effectiveness based on whether studies targeted specific personal characteristics, which encompassed several factors, such as ethnicity, geographic location, parental status, baseline body mass index, and dietary intake. The absence of differential effects may indicate that the apps are broadly effective across diverse groups, or that interventions were not sufficiently tailored to bring about subgroup specific benefits. Taken together, these findings highlight the importance of improving the reporting of markers of inequality to enable rigorous subgroup analysis. Future research should also link app-based behavioural changes to health literacy, and consider a life course approach that accounts for changing dietary needs over time, to maximise the potential impact of digital nutrition interventions.

Our results could inform the design of bespoke app-based interventions that combine BCTs and delivery techniques to facilitate changes in consumption of specific food groups. However, more work needs to be done to investigate the effects of apps on dietary consumption with direct relevance to climate goals, such as the substitution of animal-sourced foods with whole plant-based foods, as only one study included in our review addressed this. Additionally, research should evaluate the longer-term impacts of apps on behaviour change, considering how they fit within individual’s broader social and environmental contexts. This could be achieved through independent evaluations of commercial apps that have already demonstrated sustained user engagement.

### Strengths and limitations

To the author’s knowledge, this systematic review provides the most comprehensive synthesis of app-based interventions and sustainable diets to date. All studies were double screened, and data were extracted by two trained researchers, in an attempt to minimise bias and errors. A key strength is the use of three consensus-based frameworks to classify interventions, which enabled comparability to previous research and the creation of a new taxonomy that could be useful to future researchers. However, due to data availability, it was difficult to elucidate the effects of all intervention components, especially for dairy and legume outcomes.

While quantitatively synthesising effects on the intake of individual food groups is a strength of our review, inherent bias due to self-report dietary measures remains a limitation and the accuracy of our results depends on the studies’ measurement methods. Additionally, by excluding studies that reported on dietary quality or diet-related environmental impact indices, we may have missed some key literature in the field. The lack of included studies that focused specifically on sustainability meant that we could not determine whether diets were more environmentally friendly post-intervention, nor whether sustainability or health-focused content was more effective. As few studies reported on whole diets, we could not assess potential spillover effects and our classification of whether populations met dietary guidelines was limited to the food groups reported. Additionally, while our HIC focus was helpful in extending what was known from previous reviews, synthesising the evidence for LMICs will be an important next step in understanding the effective techniques that potentially differ between these settings [64, 65].

A strength of this review is the inclusion of many RCTs and the computation of a quality score that allowed comparison across study designs. Although we recognise that creating our own checklist may complicate comparisons with other reviews. Even after removing one study with high attrition from the main analysis, the overall dropout rate was still high, albeit similar to a previous review that reported 40–50% dropout across health apps [66]. Furthermore, the evidence of selection bias in some studies e.g., in gender imbalance, underscores the importance of future research recruiting more representative samples. Lastly, publication bias may have influenced the results for fruit and vegetable consumption, which emphasises the need for publishing studies with small effect sizes or null results, and systematic reviews searching grey literature more comprehensively.

## Conclusion

This review provides quantifiable evidence indicating favourable effects of apps on the consumption of fruit, vegetables, and meat. The overall changes we observed could be important for meeting recommendations for sustainable and healthy diets. We also provide actionable insights into effective BCTs and applied delivery techniques. Considering the ubiquity of apps, they have potential to influence food choice at scale, but more research is needed on the longer-term benefits and populations they work best in.

## Supplementary Information

Supplementary Material 1.

Supplementary Material 2.

## Data Availability

The datasets generated and analysed during the current study are available in the LSHTM Data Compass repository, [https://doi.org/10.17037/DATA.00004496](https:/doi.org/10.17037/DATA.00004496) [67].

## Abbreviations

BCT: Behaviour Change Technique
BCTTv1: BCT Taxonomy Version 1
BIT: Behavioural Intervention Technology
HIC: High-income country
LMIC: Low- and middle-income country
MoDO: Mode of Delivery Ontology
