# Supplementary material for: The effectiveness of mobile app-based interventions in facilitating behaviour change towards healthier and more sustainable diets: a systematic review and meta-analysis
Source: Int J Behav Nutr Phys Act. 2025 Sep 30;22:122. doi: 10.1186/s12966-025-01823-7 (PMC12487266; doi:10.1186/s12966-025-01823-7)
Supplement: Supplementary file 1 — Supplementary Material 1. [file 12966_2025_1823_MOESM1_ESM.docx]

**The effectiveness of mobile app-based interventions in facilitating behaviour change towards healthier and more sustainable diets: a systematic review and meta-analysis.**

**Additional File 1**

E Curtin, R Green, KA Brown, S Nájera Espinosa, A Chandrasekar, L Hopkins, G Turner, C Alae-Carew, K Ullian, P Scheelbeek

**Table of contents**

[Table 1. Full search strategy. 2](#_Toc206421476)

[Table 2. Inclusion and exclusion criteria. 8](#_Toc206421477)

[Table 3. High-income countries defined by the World Bank. 9](#_Toc206421478)

[Table 4. Coding of baseline participant variables. 10](#_Toc206421479)

[Table 5. National dietary recommendations used to categorise populations as meeting/not meeting dietary guidelines. 11](#_Toc206421480)

[Table 6. Definitions of intervention components. 12](#_Toc206421481)

[Table 7. Reporting transparency and risk of bias checklist. 26](#_Toc206421482)

[Table 8. Portion size estimates. 28](#_Toc206421483)

[Table 9. List of excluded studies with reasons. 29](#_Toc206421484)

[Table 10. Summary of study characteristics. 34](#_Toc206421485)

[Table 11. Summary of interventions and outcome measurements. 36](#_Toc206421486)

[Table 12. Coding framework for combinations of behaviour change techniques and delivery techniques. 37](#_Toc206421487)

[Table 13. Sensitivity analysis comparing the effects of specific and general apps. 39](#_Toc206421488)

[Table 14. Bivariate associations between exposure and outcome variables. 40](#_Toc206421489)

[Table 15. Meta-regression results for exposure and outcome variables. 41](#_Toc206421490)

[References 42](#_Toc206421491)

## **Table 1. Full search strategy.**

| **Database 1** | **Medline** |
| --- | --- |
| Search | Terms |
| 1 | (diet* or nutrition* or eat* or food or foods or meal* or portion* or grocery or groceries or recipe or cooking or (takeaway and food) or menu) adj6 (sustainab* or planetary or environmental health or environmentally friendly or pro-environment* or low-carbon or lower-carbon or reduced carbon or carbon footprint or climate or climate friendly or planet-friendly or eco-friendly or healthy or healthiness or overconsumption or processed or calorie or caloric or dairy or meat or fruits or fruit or vegetable*).mp |
| 2 | (diet* or nutrition* or eat* or food or foods or meal* or portion* or grocery or groceries or recipe or cooking or (takeaway and food) or menu).mp. |
| 3 | ((local* or seasonal*) adj3 (produce or grown or source* or food or diet)).mp. |
| 4 | conservation of natural resources/ or carbon footprint/ or climate change/ or dairy products/ or meat/ or fast foods/ |
| 5 | 2 and 3 |
| 6 | 2 and 4 |
| 7 | 1 or 5 or 6 |
| 8 | (vegetarian or vegan or pescatarian or flexitarian or lacto-ovo or plant-forward or plant-based or plant-rich or Mediterranean diet or non-dairy or EatLancet or Eat Lancet or Eatwell or Eat well or dietary guidelines or dietary recommendations).mp. |
| 9 | exp diet, Vegetarian/ or exp diet, Healthy/ or Diet Therapy/ |
| 10 | 8 or 9 |
| 11 | 7 or 10 |
| 12 | ((mobile phone* or smartphone* or smart-phone*) or ((mobile or cell or smart) adj2 (phon* or technolog* or telephon*))).mp. |
| 13 | ((mobile* or cellphone* or phone* or smartphone* or social media) adj3 (technology or app or apps or application* or intervention or game) or gamification or apps or ecological momentary intervention).mp. |
| 14 | (mHealth or m-health or eHealth or e-Health or (mobile* or phone* or smartphone* or pervasive or wireless or ubiquitous or wearable or app-based) adj2 (health)).mp. |
| 15 | (android or iPad* or iPhone*).mp. |
| 16 | ((tele) adj1 (medic* or communication* or care or caring or health)).mp. |
| 17 | ((handheld or hand-held) adj2 (technolog* or phone* or comput* or device*)).mp. |
| 18 | Gamification/ or Smartphone/ or Mobile Applications/ or Social Media/ |
| 19 | or/12-18 |
| 20 | 11 and 19 |

| **Database 2** | **Embase** |
| --- | --- |
| Search | Terms |
| 1 | (diet* or nutrition* or eat* or food or foods or meal* or portion* or grocery or groceries or recipe or cooking or (takeaway and food) or menu) adj6 (sustainab* or planetary or environmental health or environmentally friendly or pro-environment* or low-carbon or lower-carbon or reduced carbon or carbon footprint or climate or climate friendly or planet-friendly or eco-friendly or healthy or healthiness or overconsumption or processed or calorie or caloric or dairy or meat or fruits or fruit or vegetable*).mp |
| 2 | (diet* or nutrition* or eat* or food or foods or meal* or portion* or grocery or groceries or recipe or cooking or (takeaway and food) or menu).mp |
| 3 | ((local* or seasonal*) adj3 (produce or grown or source* or food or diet)).mp. |
| 4 | exp environmental sustainability/ or planetary health/ or pro-environmental behavior/ or carbon footprint/ or climate change/ or dairy product/ or meat/ or fast food/ |
| 5 | 2 and 3 |
| 6 | 2 and 4 |
| 7 | 1 or 5 or 6 |
| 8 | (vegetarian or vegan or pescatarian or flexitarian or lacto-ovo or plant-forward or plant-based or plant-rich or Mediterranean diet or non-dairy or EatLancet or Eat Lancet or Eatwell or Eat well or dietary guidelines or dietary recommendations).mp. |
| 9 | exp vegetarian diet/ or exp vegan diet/ or exp Mediterranean diet/ |
| 10 | 8 or 9 |
| 11 | 7 or 10 |
| 12 | ((mobile phone* or smartphone* or smart-phone*) or ((mobile or cell or smart) adj2 (phon* or technolog* or telephon*))).mp. |
| 13 | ((mobile* or cellphone* or phone* or smartphone* or social media) adj3 (technology or app or apps or application* or intervention or game) or gamification or apps or ecological momentary intervention).mp. |
| 14 | (mHealth or m-health or eHealth or e-Health or (mobile* or phone* or smartphone* or pervasive or wireless or ubiquitous or wearable or app-based) adj2 (health)).mp. |
| 15 | (android or iPad* or iPhone*).mp. |
| 16 | ((tele) adj1 (medic* or communication* or care or caring or health)).mp. |
| 17 | ((handheld or hand-held) adj2 (technolog* or phone* or comput* or device*)).mp. |
| 18 | gamification/ or exp mobile application/ or exp mobile phone/ or exp social media/ |
| 19 | or/12-18 |
| 20 | 11 and 19 |

| **Database 3** | **PsycINFO** |
| --- | --- |
| Search | Terms |
| 1 | (diet* or nutrition* or eat* or food or foods or meal* or portion* or grocery or groceries or recipe or cooking or (takeaway and food) or menu) adj6 (sustainab* or planetary or environmental health or environmentally friendly or pro-environment* or low-carbon or lower-carbon or reduced carbon or carbon footprint or climate or climate friendly or planet-friendly or eco-friendly or healthy or healthiness or overconsumption or processed or calorie or caloric or dairy or meat or fruits or fruit or vegetable*).mp |
| 2 | (diet* or nutrition* or eat* or food or foods or meal* or portion* or grocery or groceries or recipe or cooking or (takeaway and food) or menu).mp |
| 3 | ((local* or seasonal*) adj3 (produce or grown or source* or food or diet)).mp |
| 4 | Environmental Sustainability/ or Climate Change/ or Conservation/ or Environmental Attitudes/ or Fast Food/ |
| 5 | 2 and 3 |
| 6 | 2 and 4 |
| 7 | 1 or 5 or 6 |
| 8 | (vegetarian or vegan or pescatarian or flexitarian or lacto-ovo or plant-forward or plant-based or plant-rich or Mediterranean diet or non-dairy or EatLancet or Eat Lancet or Eatwell or Eat well or dietary guidelines or dietary recommendations).mp. |
| 9 | 7 or 8 |
| 10 | ((mobile phone* or smartphone* or smart-phone*) or ((mobile or cell or smart) adj2 (phon* or technolog* or telephon*))).mp |
| 11 | ((mobile* or cellphone* or phone* or smartphone* or social media) adj3 (technology or app or apps or application* or intervention or game) or gamification or apps or ecological momentary intervention).mp |
| 12 | (mHealth or m-health or (mobile* or phone* or smartphone* or pervasive or wireless or ubiquitous or wearable or app-based) adj2 (health)).mp |
| 13 | (android or iPad* or iPhone*).mp. |
| 14 | ((tele) adj1 (medic* or communication* or care or caring or health)).mp. |
| 15 | ((handheld or hand-held) adj2 (technolog* or phone* or comput* or device*)).mp. |
| 16 | exp Mobile Phones/ or Mobile Applications/ or Social Media/ |
| 17 | or/10-16 |
| 18 | 9 and 17 |

| **Database 4** | **Global Health** |
| --- | --- |
| Search | Terms |
| 1 | (diet* or nutrition* or eat* or food or foods or meal* or portion* or grocery or groceries or recipe or cooking or (takeaway and food) or menu) adj6 (sustainab* or planetary or environmental health or environmentally friendly or pro-environment* or low-carbon or lower-carbon or reduced carbon or carbon footprint or climate or climate friendly or planet-friendly or eco-friendly or healthy or healthiness or overconsumption or processed or calorie or caloric or dairy or meat or fruits or fruit or vegetable*).mp |
| 2 | (diet* or nutrition* or eat* or food or foods or meal* or portion* or grocery or groceries or recipe or cooking or (takeaway and food) or menu).mp |
| 3 | ((local* or seasonal*) adj3 (produce or grown or source* or food or diet)).mp |
| 4 | climate change/ or environmental health/ or exp environmental impact/ or milk products/ or processed foods/ or meat/ or animal products/ |
| 5 | 2 and 3 |
| 6 | 2 and 4 |
| 7 | 1 or 5 or 6 |
| 8 | (vegetarian or vegan or pescatarian or flexitarian or lacto-ovo or plant-forward or plant-based or plant-rich or Mediterranean diet or non-dairy or EatLancet or Eat Lancet or Eatwell or Eat well or dietary guidelines or dietary recommendations).mp. |
| 9 | vegetarians/ or vegetarianism/ or vegetarian diets/ or vegans/ or plant products/ or Mediterranean diet/ |
| 10 | 8 or 9 |
| 11 | 7 or 10 |
| 12 | ((mobile phone* or smartphone* or smart-phone*) or ((mobile or cell or smart) adj2 (phon* or technolog* or telephon*))).mp. |
| 13 | ((mobile* or cellphone* or phone* or smartphone* or social media) adj3 (technology or app or apps or application* or intervention or game) or gamification or apps or ecological momentary intervention).mp |
| 14 | (mHealth or m-health or (mobile* or phone* or smartphone* or pervasive or wireless or ubiquitous or wearable or app-based) adj2 (health)).mp. |
| 15 | (android or iPad* or iPhone*).mp. |
| 16 | ((tele) adj1 (medic* or communication* or care or caring or health)).mp. |
| 17 | ((handheld or hand-held) adj2 (technolog* or phone* or comput* or device*)).mp. |
| 18 | exp mobile telephones/ or mobile applications/ or mHealth/ or m-health/ or exp social media/ |
| 19 | or/12-18 |
| 20 | 11 and 19 |

| **Database 5** | **CINAHL** |
| --- | --- |
| Search | Terms |
| 1 | (diet* or nutrition* or eat* or food or foods or meal* or portion* or grocery or groceries or recipe or cooking or (takeaway and food) or menu) N6 (sustainab* or planetary or “environmental health” or “environmentally friendly” or pro-environment* or low-carbon or lower-carbon or “reduced carbon” or “carbon footprint” or “climate friendly” or climate or eco-friendly or planet-friendly or healthy or healthiness or overconsumption or processed or calorie or caloric or dairy or meat or fruits or fruit or vegetable*) |
| 2 | (diet* or nutrition* or eat* or food or foods or meal* or portion* or grocery or groceries or recipe or cooking or (takeaway and food) or menu) |
| 3 | ((local* or seasonal*) N3 (grown or source* or food or produce or diet)) |
| 4 | (MH “Carbon Footprint”) or (MH “Environmental Sustainability”) or (MH “Dairy Products”) or (MH “Milk Substitutes”) or (MH “Meat”) |
| 5 | S2 AND S3 |
| 6 | S2 AND S4 |
| 7 | S1 OR S5 OR S6 |
| 8 | (vegetarian or vegan or pescatarian or flexitarian or lacto-ovo or plant-forward or plant-based or plant-rich or “Mediterranean diet” or non-dairy or EatLancet or “Eat Lancet” or Eatwell or “Eat well” or “dietary guidelines” or “dietary recommendations”) |
| 9 | (MH “Vegetarianism”) or (MH “Mediterranean Diet”) or (MH “Diet, Western”) or (MH “Plant-Based Diet”) or (MH “Nutritional Requirements+”) |
| 10 | S8 OR S9 |
| 11 | S7 OR S10 |
| 12 | ((mobile phone* or smartphone* or smart-phone*) or ((mobile or cell or smart) N2 (phon* or technolog* or telephon*))) |
| 13 | ((mobile* or cellphone* or phone* or smartphone* or “social media”) N3 (technology or app or apps or application* or intervention or game) or gamification or apps or “ecological momentary intervention”)) |
| 14 | (mHealth or m-health or (mobile* or phone* or smartphone* or pervasive or wireless or ubiquitous or wearable or app-based) N2 (health)) |
| 15 | (android or iPad* or iPhone*) |
| 16 | ((tele) N1 (medic* or communication* or care or caring or health)) |
| 17 | ((handheld or hand-held) N2 (technolog* or phone* or comput* or device*)) |
| 18 | (MH “Cellular Phone”) or (MH “Smartphone”) or (MH “Social Media+”) |
| 19 | S12 OR S13 OR S14 OR S15 OR S16 OR S17 OR S18 |
| 20 | S11 AND S19 |

| **Database 6** | **GreenFILE** |
| --- | --- |
| Search | Terms |
| 1 | (diet* or nutrition* or eat* or food or foods or meal* or portion* or grocery or groceries or recipe or cooking or (takeaway and food) or menu) N6 (sustainab* or planetary or “environmental health” or “environmentally friendly” or pro-environment* or low-carbon or lower-carbon or “reduced carbon” or “carbon footprint” or “climate friendly” or climate or eco-friendly or planet-friendly or healthy or healthiness or overconsumption or processed or calorie or caloric or dairy or meat or fruits or fruit or vegetable*) |
| 2 | (diet* or nutrition* or eat* or food or foods or meal* or portion* or grocery or groceries or recipe or cooking or (takeaway and food) or menu) |
| 3 | ((local* or seasonal*) N3 (grown or source* or food or produce or diet)) |
| 4 | (DE "SUSTAINABILITY" OR DE "SUSTAINABLE communities" or DE "ENVIRONMENTAL psychology" OR DE "ATTITUDES toward the environment" or DE "ENVIRONMENTAL responsibility") |
| 5 | S2 AND S3 |
| 6 | S2 AND S4 |
| 7 | S1 OR S5 OR S6 |
| 8 | (vegetarian or vegan or pescatarian or flexitarian or lacto-ovo or plant-forward or plant-based or plant-rich or “Mediterranean diet” or non-dairy or EatLancet or “Eat Lancet” or Eatwell or “Eat well” or “dietary guidelines” or “dietary recommendations”) |
| 9 | (DE "VEGETARIAN foods" OR DE "VEGETARIAN restaurants" OR DE "VEGETARIANISM" OR DE "VEGETARIANS" OR DE "VEGANS" OR DE "PLANT-based diet") |
| 10 | S8 OR S9 |
| 11 | S7 OR S10 |
| 12 | ((mobile phone* or smartphone* or smart-phone*) or ((mobile or cell or smart) N2 (phon* or technolog* or telephon*))) |
| 13 | ((mobile* or cellphone* or phone* or smartphone* or “social media”) N3 (technology or app or apps or application* or intervention or game) or gamification or apps or “ecological momentary intervention”)) |
| 14 | (mHealth or m-health or (mobile* or phone* or smartphone* or pervasive or wireless or ubiquitous or wearable or app-based) N2 (health)) |
| 15 | (android or iPad* or iPhone*) |
| 16 | ((tele) N1 (medic* or communication* or care or caring or health)) |
| 17 | ((handheld or hand-held) N2 (technolog* or phone* or comput* or device*)) |
| 18 | S12 OR S13 OR S14 OR S15 OR S16 OR S17 |
| 20 | S11 AND S18 |

| **Database 7** | **Web of Science** |
| --- | --- |
| Search | Terms |
| 1 | TS=((diet* or nutrition* or eat* or food or foods or meal* or portion* or grocery or groceries or recipe or cooking or menu) NEAR/6 (sustainab* or planetary or “environmental health” or “environmentally friendly” or pro-environment* or low-carbon or lower-carbon or “reduced carbon” or “carbon footprint” or “climate friendly” or climate or eco-friendly or planet-friendly or healthy or healthiness or overconsumption or processed or calorie or caloric or dairy or meat or fruits or fruit or vegetable*)) OR TS=((diet* or nutrition* or eat* or food or foods or meal* or portion* or grocery or groceries or recipe or cooking or (takeaway and food) or menu) AND ((local* or seasonal*) NEAR/3 (grown or source* or food or produce or diet)) |
| 2 | TS=(vegetarian or vegan or pescatarian or flexitarian or lacto-ovo or plant-forward or plant-based or plant-rich or “Mediterranean diet” or non-dairy or EatLancet or “Eat Lancet” or Eatwell or “Eat well” or “dietary guidelines” or “dietary recommendations”) |
| 3 | (TS=((mobile phone* or smartphone* or smart-phone*) or ((mobile or cell or smart)) NEAR/2 (phon* or technolog* or telephon*)) OR TS=(((mobile* or cellphone* or phone* or smartphone* or “social media”) NEAR/3 (technology or app or apps or application* or intervention or game) or gamification or apps or “ecological momentary intervention”) OR TS=((mHealth or m-health or (mobile* or phone* or smartphone* or pervasive or wireless or ubiquitous or wearable or app-based)) NEAR/2 (health)) OR TS=(android or iPad* or iPhone*) OR TS=(((tele) NEAR/1 (medic* or communication* or care or caring or health))) OR TS=((handheld or hand-held) NEAR/2 (technolog* or phone* or comput* or device*))) |
| 4 | #2 OR #1 |
| 5 | #3 AND #4 |

| **Registry 1** | **Clinicaltrials.gov** |
| --- | --- |
|  | (diet OR nutrition OR eat OR food OR meal OR portion) AND (sustainability OR planetary OR environmental health OR pro-environment OR low-carbon OR climate change OR healthy OR calorie OR dairy OR meat OR fruit OR vegetable)  AND  (mobile phone OR cellphone OR smartphone OR social media OR app OR apps OR gamification OR ecological momentary intervention)  Filters:  Age adults (18-64) or older adults (65+)  Completed |
| **Registry 2** | **Cochrane Central Register of Controlled Trials (CENTRAL)** |
| Search | Terms |
| 1 | (diet* or nutrition* or eat* or food or foods or meal* or portion* or grocery or groceries or recipe or cooking or menu) NEAR/6 (sustainab* or planetary or “environmental health” or “environmentally friendly” or pro-environment* or low-carbon or lower-carbon or “reduced carbon” or “carbon footprint” or “climate friendly” or climate or eco-friendly or planet-friendly or healthy or healthiness or overconsumption or processed or calorie or caloric or dairy or meat or fruits or fruit or vegetable*) |
| 2 | (diet* or nutrition* or eat* or food or foods or meal* or portion* or grocery or groceries or recipe or cooking or (takeaway and food) or menu) AND ((local* or seasonal*) NEAR/3 (grown or source* or food or produce or diet)) OR (vegetarian or vegan or pescatarian or flexitarian or lacto-ovo or plant-forward or plant-based or plant-rich or “Mediterranean diet” or non-dairy or EatLancet or “Eat Lancet” or Eatwell or “Eat well” or “dietary guidelines” or “dietary recommendations”) |
| 3 | (mobile phone* or smartphone* or smart-phone*) OR ((mobile or cell or smart) AND (phon* or technolog* or telephon*)) OR ((mobile* or cellphone* or phone* or smartphone* or “social media”) AND (technology or app or apps or application* or intervention or game) or gamification or apps or “ecological momentary intervention”) OR ((mHealth or m-health or (mobile* or phone* or smartphone* or pervasive or wireless or ubiquitous or wearable or app-based)) AND (health)) OR (android or iPad* or iPhone*) OR ((tele) AND (medic* or communication* or care or caring or health)) OR ((handheld or hand-held) AND (technolog* or phone* or comput* or device*)) |

|  | **Google Chrome** |
| --- | --- |
| Search | Terms |
| 1 | (mobile app) and (diet or nutrition or food) and (behaviour change) |

| PICOTS | Inclusion criteria | Exclusion criteria |
| --- | --- | --- |
| Population | Adults (18+ y).  No health conditions.  Clinically healthy (including overweight or obese). | Non-adult population.  Specific nutritional requirements, e.g., pregnant women or those with previous health conditions. |
| Intervention | Incorporate a least one app-only arm or app + smartwatch.  App is for personal use.  App is delivered independently from health professionals. | App is one component of a multi-component intervention, a secondary way to access website content, only used for assessment, or only for delivery of professional healthcare support. |
| Comparator | No intervention control, a different/less intensive intervention, or baseline measure. | No comparator or control measure. |
| Outcome | Actual consumption (dietary intake or purchases) of one of the following six food categories: fruit and vegetables, legumes, nuts, fish, dairy, or meat.  Measured objectively (e.g., direct observation, weight before and after consumption) or subjectively (e.g., dietary recalls or questionnaires).  Data are reported in a numeric scale suitable for meta-analysis.  Defined at population, household, or individual level. | Pre-consumption indicators, e.g., intention or willingness.  Post-consumption indicators, e.g., nutrient status, biomarkers, or anthropometrics.  Intake of a specific nutrient.  Food waste. |
| Type of study | Randomised controlled trials, non-randomised trials, and pre/post designs.  Written in the English language. | Cross-sectional study, review/meta-analysis, or qualitative study. |
| Situation | High-income countries as defined by the World Bank. | Low- or middle-income countries. |

## **Table 2. Inclusion and exclusion criteria.**

## **Table 3. High-income countries defined by the World Bank.**

Aruba

Andorra

United Arab Emirates

Antigua and Barbuda

Australia

Austria

Belgium

Bahrain

The Bahamas

Bermuda

Barbados

Brunei Darussalam

Canada

Switzerland

Channel Islands

Chile

Curaçao

Cayman Islands

Cyprus

Czech Republic

Germany

Denmark

Spain

Estonia

Finland

France

Faroe Islands

United Kingdom

Gibraltar

Greece

Greenland

Guam

Hong Kong SAR, China

Croatia

Hungary

Isle of Man

Ireland

Iceland

Israel

Italy

Japan

St. Kitts and Nevis

Korea, Rep.

Kuwait

Liechtenstein

Lithuania

Luxembourg

Latvia

Macao SAR, China

St. Martin (French part)

Monaco

Malta

Northern Mariana Islands

New Caledonia

Netherlands

Norway

Nauru

New Zealand

Oman

Panama

Poland

Puerto Rico

Portugal

French Polynesia

Qatar

Romania

Saudi Arabia

Singapore

San Marino

Slovak Republic

Slovenia

Sweden

Sint Maarten (Dutch part)

Seychelles

Turks and Caicos Islands

Trinidad and Tobago

Taiwan, China

Uruguay

United States

British Virgin Islands

Virgin Islands (U.S.)

## **Table 4. Coding of baseline participant variables.**

| Study | Country | Target population | Participant type | Baseline daily consumption | Dietary recommendations |
| --- | --- | --- | --- | --- | --- |
| Appleton et al., 2019 | UK | Younger adults | General | 3.4 (FV) | Not met |
| Aulbach et al., 2021 | UK | General population | General | 1.52 (FV) | Not met |
| Bhurosy et al., 2020 | USA | General population | General | 0.95 (FV) | Not met |
| Brewer et al., 2019 | USA | African Americans with cardiovascular disease risk factors | Specific | 3.4 (FV) | Not met |
| Carfora and Catellani, 2022 | Italy | General population | General | 0.26 (legumes), 0.95 (meat) | Not met |
| Chung et al., 2021 | China | General population | General | 7 (FV) | Met |
| Eisenhauer et al., 2021 | USA | Rural OW/OB men | Specific | 2.26 (FV) | Not met |
| Elbert et al., 2016 | Netherlands | Adults with inadequate FV intake | Specific | 1.97 (fruit) | Not met |
| Gonzalez-Ramirez et al., 2022 | Spain | Younger adults | General | 3.09 (FV), 0.09 (legumes),  0.56 (meat) | Not met |
| Hahn et al., 2021 | USA | Female students | General | 2.79 (FV) | Not met |
| Hendrie et al., 2020 | Australia | General population | General | 3.06 (vegetables) | Met |
| Inauen et al., 2017 | Switzerland | General population | General | 4.4 (FV) | Not met |
| Kliemann et al., 2019 | UK | OW/OB adults | Specific | 1.65 (FV) | Not met |
| Mummah et al., 2016 | USA | OW/OB adults | Specific | 6.5 (FV) | Met |
| Mummah et al., 2017 | USA | OW/OB adults | Specific | 4.65 (FV) | Met |
| Nezami et al., 2022 | USA | OW/OB parents | Specific | 2.18 (FV) | Not met |
| Palacios et al., 2018 | Puerto Rico | Hispanic OW/OB adults | Specific | 1.71 (FV) | Not met |
| Price et al., 2020 | UK | General population | General | 3.12 (FV) | Not met |
| Recio-Rodriguez et al., 2018 | Spain | General population | General | 7.29 (FV), 0.17 (legumes),  0.45 (meat) | Not met |
| Wilson et al., 2023 | New Zealand | Airline pilots | General | 4.5 (FV) | Not met |

OW/OB: overweight or obese; FV: fruit and vegetables.

## **Table 5. National dietary recommendations used to categorise populations as meeting/not meeting dietary guidelines.**

|  | Recommended number of daily portions in each country |
| --- | --- |
| Fruit and vegetables |  |
| UK | 5 ^1^ |
| USA | 4.5 ^a^ ^2^ |
| China | 5.5-9.5 ^b^ ^3^ |
| The Netherlands | 5 ^c^ ^4^ |
| Spain | 5 ^4^ |
| Australia | 5 ^5^ |
| Switzerland | 5 ^4^ |
| Puerto Rico | 4.5 ^2^ |
| New Zealand | 7-8 ^d^ ^6^ |
| Legumes |  |
| Italy | 0.43 ^e^ ^7^ |
| Spain | 0.57 ^f^ ^7^ |
| Meat |  |
| Italy | 0.29-0.57 ^g^ ^8^ |
| Spain | 0.43 ^h^ ^8^ |

^a^ Recommended fruit and vegetables in cup eq/day for an adult consuming 2000kcal per day.

^b^ 500-850g fruit and vegetables per day (200-350g fruit and 300-500g vegetables).

^c^ 450g fruit and vegetables per day (200g fruit and 250g vegetables).

^d^ 2 portions fruit and 5-6 portions vegetables per day.

^e^ 3 portions legumes per week.

^f^ 4 portions legumes per week.

^g^ 2-4 portions total meat per week (1 portion red meat and 1-3 portions white meat).

^h^ 3 portions total meat per week (this is a maximum; the guidelines are to consume 0-3 portions).

## **Table 6. Definitions of intervention components.**

| **Reference** | **Name** | **Description** | **Example** |
| --- | --- | --- | --- |
| **BCTs** |  | | |
| **1** | **Goals and planning** | | |
| **1.1** | Goal setting (behavior) | Set or agree on a goal defined in terms of the behavior to be achieved | Agree on a daily walking goal (e.g. 3 miles) with the person and reach agreement about the goal. Set the goal of eating 5 pieces of fruit per day as specified in public health guidelines. |
| **1.2** | Problem solving | Analyse, or prompt the person to analyse, factors influencing the behavior and generate or select strategies that include overcoming barriers and/or increasing facilitators (includes 'Relapse Prevention' and 'Coping Planning') Note: barrier identification without solutions is not sufficient. If the BCT does not include analysing the behavioral problem, consider 12.3, Avoidance/changing exposure to cues for the behavior, 12.1, Restructuring the physical environment, 12.2, Restructuring the social environment, or 11.2, Reduce negative emotions | Identify specific triggers (e.g. being in a pub, feeling anxious) that generate the urge/want/need to drink and develop strategies for avoiding environmental triggers or for managing negative emotions, such as anxiety, that motivate drinking |
| **1.3** | Goal setting (outcome) | Set or agree on a goal defined in terms of a positive outcome of wanted behavior | Set a weight loss goal (e.g. 0.5 kilogram over one week) as an outcome of changed eating patterns |
| **1.4** | Action planning | Prompt detailed planning of performance of the behavior (must include at least one of context, frequency, duration and intensity). Context may be environmental (physical or social) or internal (physical, emotional or cognitive) (includes 'Implementation Intentions') Note: evidence of action planning does not necessarily imply goal setting, only code latter if sufficient evidence | Encourage a plan to carry condoms when going out socially at weekends Prompt planning the performance of a particular physical activity (e.g. running) at a particular time (e.g. before work) on certain days of the week |
| **1.5** | Review behavior goal(s) | Review behavior goal(s) jointly with the person and consider modifying goal(s) or behavior change strategy in light of achievement. This may lead to re-setting the same goal, a small change in that goal or setting a new goal instead of (or in addition to) the first, or no change Note: if goal specified in terms of behavior, code 1.5, Review behavior goal(s), if goal unspecified, code 1.7, Review outcome goal(s); if discrepancy created consider also 1.6, Discrepancy between current behavior and goal | Examine how well a person's performance corresponds to agreed goals e.g. whether they consumed less than one unit of alcohol per day, and consider modifying future behavioral goals accordingly e.g. by increasing or decreasing alcohol target or changing type of alcohol consumed |
| **1.6** | Discrepancy between current behavior and goal | Draw attention to discrepancies between a person's current behavior (in terms of the form, frequency, duration, or intensity of that behavior) and the person's previously set outcome goals, behavioral goals or action plans (goes beyond self-monitoring of behavior) Note: if discomfort is created only code 13.3, Incompatible beliefs and not 1.6, Discrepancy between current behavior and goal; if goals are modified, also code 1.5, Review behavior goal(s) and/or 1.7, Review outcome goal(s); if feedback is provided, also code 2.2, Feedback on behaviour | Point out that the recorded exercise fell short of the goal set |
| **1.7** | Review outcome goal(s) | Review outcome goal(s) jointly with the person and consider modifying goal(s) in light of achievement. This may lead to re-setting the same goal, a small change in that goal or setting a new goal instead of, or in addition to the first Note: if goal specified in terms of behavior, code 1.5, Review behavior goal(s), if goal unspecified, code 1.7, Review outcome goal(s); if discrepancy created consider also 1.6, Discrepancy between current behavior and goal | Examine how much weight has been lost and consider modifying outcome goal(s) accordingly e.g., by increasing or decreasing subsequent weight loss targets |
| **1.8** | Behavioral contract | Create a written specification of the behavior to be performed, agreed on by the person, and witnessed by another Note: also code 1.1, Goal setting (behavior) | Sign a contract with the person e.g. specifying that they will not drink alcohol for one week |
| **1.9** | Commitment | Note: if defined in terms of the behavior to be achieved also code 1.1, Goal setting (behavior) | Ask the person to use an 'I will' statement to affirm or reaffirm a strong commitment (i.e. using the words strongly, committed, or high priority) to start, continue or restart the attempt to take medication as prescribed |
|  |  |  |  |
| **2** | **Feedback and monitoring** | | |
| **2.1** | Monitoring of behavior by others without feedback | Observe or record behavior with the person's knowledge as part of a behavior change strategy Note: if monitoring is part of a data collection procedure rather than a strategy aimed at changing behavior, do not code; if feedback given, code only 2.2, Feedback on behavior, and not 2.1, Monitoring of behavior by others with feedback; if monitoring outcome(s) code 2.5, Monitoring outcome(s) of behavior by others without feedback; if self-monitoring behavior, code 2.3, Self-monitoring of behaviour | Watch hand washing behaviors among health care staff and make notes on context, frequency and technique used |
| **2.2** | Monitoring of behavior by others with feedback | Monitor and provide informative or evaluative feedback on performance of the behavior (e.g. form, frequency, duration, intensity) Note: if Biofeedback, code only 2.6, Biofeedback and not 2.2, Feedback on behavior; if feedback is on outcome(s) of behavior, code 2.7, Feedback on outcome(s) of behavior; if there is no clear evidence that feedback was given, code 2.1, Monitoring of behavior by others without feedback; if feedback on behaviour is evaluative e.g. praise, also code 10.4, Social reward | Inform the person of how many steps they walked each day (as recorded on a pedometer) or how many calories they ate each day (based on a food consumption questionnaire) |
| **2.3** | Self-monitoring of behavior | Establish a method for the person to monitor and record their behavior(s) as part of a behavior change strategy Note: if monitoring is part of a data collection procedure rather than a strategy aimed at changing behavior, do not code; if monitoring of outcome of behavior, code 2.4, Self-monitoring of outcome(s) of behavior; if monitoring is by someone else (without feedback), code 2.1, Monitoring of behavior by others without feedback | Ask the person to record daily, in a diary, whether they have brushed their teeth for at least two minutes before going to bed Give patient a pedometer and a form for recording daily total number of steps |
| **2.4** | Self-monitoring of outcome(s) of behavior | Establish a method for the person to monitor and record the outcome(s) of their behavior as part of a behavior change strategy Note: if monitoring is part of a data collection procedure rather than a strategy aimed at changing behavior, do not code ; if monitoring behavior, code 2.3, Self-monitoring of behavior; if monitoring is by someone else (without feedback), code 2.5, Monitoring outcome(s) of behavior by others without feedback | Ask the person to weigh themselves at the end of each day, over a two week period, and record their daily weight on a graph to increase exercise behaviors |
| **2.5** | Monitoring outcome(s) of behavior by others without feedback | Observe or record outcomes of behavior with the person's knowledge as part of a behavior change strategy Note: if monitoring is part of a data collection procedure rather than a strategy aimed at changing behavior, do not code; if feedback given, code only 2.7, Feedback on outcome(s) of behavior; if monitoring behavior code 2.1, Monitoring of behavior by others without feedback; if self-monitoring outcome(s), code 2.4, Self-monitoring of outcome(s) of behavior | Record blood pressure, blood glucose, weight loss, or physical fitness |
| **2.6** | Biofeedback | Provide feedback about the body (e.g. physiological or biochemical state) using an external monitoring device as part of a behavior change strategy Note: if Biofeedback, code only 2.6, Biofeedback and not 2.2, Feedback on behavior or 2.7, Feedback on outcome(s) of behaviour | Inform the person of their blood pressure reading to improve adoption of health behaviors |
| **2.7** | Feedback on outcome(s) of behavior | Monitor and provide feedback on the outcome of performance of the behavior Note: if Biofeedback, code only 2.6, Biofeedback and not 2.7, Feedback on outcome(s) of behavior; if feedback is on behavior code 2.2, Feedback on behavior; if there is no clear evidence that feedback was given code 2.5, Monitoring outcome(s) of behavior by others without feedback; if feedback on behaviour is evaluative e.g. praise, also code 10.4, Social reward | Inform the person of how much weight they have lost following the implementation of a new exercise regime |
|  |  |  |  |
| **3** | **Social support** | |  |
| **3.1** | Social support (unspecified) | Advise on, arrange or provide social support (e.g. from friends, relatives, colleagues,' buddies' or staff) or non-contingent praise or reward for performance of the behavior. It includes encouragement and counselling, but only when it is directed at the behavior | Advise the person to call a 'buddy' when they experience an urge to smoke Arrange for a housemate to encourage continuation with the behavior change programme Give information about a self-help group that offers support for the behavior |
| **3.2** | Social support (practical) | Advise on, arrange, or provide practical help (e.g. from friends, relatives, colleagues, 'buddies' or staff) for performance of the behavior  Note: if emotional, code 3.3, Social support (emotional); if general or unspecified, code 3.1, Social support (unspecified) If only restructuring the physical environment or adding objects to the environment, code 12.1, Restructuring the physical environment or 12.5, Adding objects to the environment; attending a group or class and/or mention of 'follow-up' does not necessarily apply this BCT, support must be explicitly mentioned. | Ask the partner of the patient to put their tablet on the breakfast tray so that the patient remembers to take it |
| **3.3** | Social support (emotional) | Advise on, arrange, or provide emotional social support (e.g. from friends, relatives, colleagues, 'buddies' or staff) for performance of the behavior Note: if practical, code 3.2, Social support (practical); if unspecified, code 3.1, Social support (unspecified) | Ask the patient to take a partner or friend with them to their colonoscopy appointment |
|  |  |  |  |
| **4** | **Shaping knowledge** | |  |
| **4.1** | Instruction on how to perform a behavior | Advise or agree on how to perform the behavior (includes 'Skills training') Note: when the person attends classes such as exercise or cookery, code 4.1, Instruction on how to perform the behavior, 8.1, Behavioral practice/rehearsal and 6.1, Demonstration of the behavior | Advise the person how to put a condom on a model of a penis correctly |
| **4.2** | Information about antecedents | Provide information about antecedents (e.g. social and environmental situations and events, emotions, cognitions) that reliably predict performance of the behaviour | Advise to keep a record of snacking and of situations or events occurring prior to snacking |
| **4.3** | Re-attribution | Elicit perceived causes of behavior and suggest alternative explanations (e.g. external or internal and stable or unstable) | If the person attributes their over-eating to the frequent presence of delicious food, suggest that the 'real' cause may be the person's inattention to bodily signals of hunger and satiety |
| **4.4** | Behavioral experiments | Advise on how to identify and test hypotheses about the behavior, its causes and consequences, by collecting and interpreting data | Ask a family physician to give evidence-based advice rather than prescribe antibiotics and to note whether the patients are grateful or annoyed |
|  |  |  |  |
| **5** | **Natural consequences** | |  |
| **5.1** | Information about health consequences | Provide information (e.g. written, verbal, visual) about health consequences of performing the behavior Note: consequences can be for any target, not just the recipient(s) of the intervention; emphasising importance of consequences is not sufficient; if information about emotional consequences, code 5.6, Information about emotional consequences; if about social, environmental or unspecified consequences code 5.3, Information about social and environmental consequences | Explain that not finishing a course of antibiotics can increase susceptibility to future infection Present the likelihood of contracting a sexually transmitted infection following unprotected sexual behavior |
| **5.2** | Salience of consequences | Use methods specifically designed to emphasise the consequences of performing the behaviour with the aim of making them more memorable (goes beyond informing about consequences) Note: if information about consequences, also code 5.1, Information about health consequences, 5.6, Information about emotional consequences or 5.3, Information about social and environmental consequences | Produce cigarette packets showing pictures of health consequences e.g. diseased lungs, to highlight the dangers of continuing to smoke |
| **5.3** | Information about social and environmental consequences | Provide information (e.g. written, verbal, visual) about social and environmental consequences of performing the behavior Note: consequences can be for any target, not just the recipient(s) of the intervention; if information about health or consequences, code 5.1, Information about health consequences; if about emotional consequences, code 5.6, Information about emotional consequences; if unspecified, code 5.3, Information about social and environmental consequences | Tell family physician about financial remuneration for conducting health screening Inform a smoker that the majority of people disapprove of smoking in public places |
| **5.4** | Monitoring of emotional consequences | Prompt assessment of feelings after attempts at performing the behavior | Agree that the person will record how they feel after taking their daily walk |
| **5.5** | Anticipated regret | Induce or raise awareness of expectations of future regret about performance of the unwanted behavior Note: not including 5.6, Information about emotional consequences if suggests adoption of a perspective or new perspective in order to change cognitions also code 13.2, Framing/reframing Ask the person to assess the degree of regret they will feel if they do not quit smoking | Ask the person to assess the degree of regret they will feel if they do not quit smoking |
| **5.6** | Information about emotional consequences | Provide information (e.g. written, verbal, visual) about emotional consequences of performing the behavior Note: consequences can be related to emotional health disorders (e.g. depression, anxiety) and/or states of mind (e.g. low mood, stress); not including 5.5, Anticipated regret; consequences can be for any target, not just the recipient(s) of the intervention; if information about health consequences code 5.1, Information about health consequences; if about social, environmental or unspecified code 5.3, Information about social and environmental consequences | Explain that quitting smoking increases happiness and life satisfaction |
|  |  |  |  |
| **6** | **Comparison of behaviour** | |  |
| **6.1** | Demonstration of the behavior | Provide an observable sample of the performance of the behaviour, directly in person or indirectly e.g. via film, pictures, for the person to aspire to or imitate (includes'Modelling'). Note: if advised to practice, also code, 8.1, Behavioural practice and rehearsal; If provided with instructions on how to perform, also code 4.1, Instruction on how to perform the behaviour | Demonstrate to nurses how to raise the issue of excessive drinking with patients via a role-play exercise |
| **6.2** | Social comparison | Draw attention to others' performance to allow comparison with the person's own performance  Note: being in a group setting does not necessarily mean that social comparison is actually taking place | Show the doctor the proportion of patients who were prescribed antibiotics for a common cold by other doctors and compare with their own data |
| **6.3** | Information about others' approval | Provide information about what other people think about the behavior. The information clarifies whether others will like, approve or disapprove of what the person is doing or will do | Tell the staff at the hospital ward that staff at all other wards approve of washing their hands according to the guidelines |
|  |  |  |  |
| **7** | **Associations** | |  |
| **7.1** | Prompts/cues | Introduce or define environmental or social stimulus with the purpose of prompting or cueing the behavior. The prompt or cue would normally occur at the time or place of performance  Note: when a stimulus is linked to a specific action in an if-then plan including one or more of frequency, duration or intensity also code 1.4, Action planning. | Put a sticker on the bathroom mirror to remind people to brush their teeth |
| **7.2** | Cue signalling reward | Identify an environmental stimulus that reliably predicts that reward will follow the behavior (includes 'Discriminative cue') | Advise that a fee will be paid to dentists for a particular dental treatment of 6-8 year old, but not older, children to encourage delivery of that treatment (the 6-8 year old children are the environmental stimulus) |
| **7.3** | Reduce prompts/cues | Withdraw gradually prompts to perform the behavior (includes 'Fading') | Reduce gradually the number of reminders used to take medication |
| **7.4** | Remove access to the reward | Advise or arrange for the person to be separated from situations in which unwanted behavior can be rewarded in order to reduce the behavior (includes 'Time out') | Arrange for cupboard containing high calorie snacks to be locked for a specified period to reduce the consumption of sugary foods in between meals |
| **7.5** | Remove aversive stimulus | Advise or arrange for the removal of an aversive stimulus to facilitate behavior change (includes 'Escape learning') | Arrange for a gym-buddy to stop nagging the person to do more exercise in order to increase the desired exercise behaviour |
| **7.6** | Satiation | Advise or arrange repeated exposure to a stimulus that reduces or extinguishes a drive for the unwanted behavior | Arrange for the person to eat large quantities of chocolate, in order to reduce the person's appetite for sweet foods |
| **7.7** | Exposure | Provide systematic confrontation with a feared stimulus to reduce the response to a later encounter | Agree a schedule by which the person who is frightened of surgery will visit the hospital where they are scheduled to have surgery |
| **7.8** | Associative learning | Present a neutral stimulus jointly with a stimulus that already elicits the behavior repeatedly until the neutral stimulus elicits that behavior (includes 'Classical/Pavlovian Conditioning') Note: when a BCT involves reward or punishment, code one or more of: 10.2, Material reward (behavior); 10.3, Non-specific reward; 10.4, Social reward, 10.9, Self-reward; 10.10, Reward (outcome) | Present repeatedly fatty foods with a disliked sauce to discourage the consumption of fatty foods |
|  |  |  |  |
| **8** | **Repetition and substitution** | |  |
| **8.1** | Behavioral practice/ rehearsal | Prompt practice or rehearsal of the performance of the behaviour one or more times in a context or at a time when the performance may or may not be necessary, in order to increase habit and skill Note: if aiming to associate performance with the context, also code 8.3, Habit formation | Prompt asthma patients to practice measuring their peak flow in the nurse's consulting room |
| **8.2** | Behavior substitution | Prompt substitution of the unwanted behavior with a wanted or neutral behavior Note: if this occurs regularly, also code 8.4, Habit reversal | Suggest that the person goes for a walk rather than watches television |
| **8.3** | Habit formation | Prompt rehearsal and repetition of the behavior in the same context repeatedly so that the context elicits the behavior | Prompt patients to take their statin tablet before brushing their teeth every evening |
| **8.4** | Habit reversal | Prompt rehearsal and repetition of an alternative behavior to replace an unwanted habitual behavior Note: also code 8.2, Behavior substitution | Ask the person to walk up stairs at work where they previously always took the lift |
| **8.5** | Overcorrection | Ask to repeat the wanted behavior in an exaggerated way following an unwanted behaviour | Ask to eat only fruit and vegetables the day after a poor diet |
| **8.6** | Generalisation of a target behavior | Advise to perform the wanted behaviour, which is already performed in a particular situation, in another situation | Advise to repeat toning exercises learned in the gym when at home |
| **8.7** | Graded tasks | Set easy-to-perform tasks, making them increasingly difficult, but achievable, until behavior is performed | Ask the person to walk for 100 yards a day for the first week, then half a mile a day after they have successfully achieved 100 yards, then two miles a day after they have successfully achieved one mile |
|  |  |  |  |
| **9** | **Comparison of outcomes** | |  |
| **9.1** | Credible source | Present verbal or visual communication from a credible source in favour of or against the behavior Note: code this BCT if source generally agreed on as credible e.g., health professionals, celebrities or words used to indicate expertise or leader in field and if the communication has the aim of persuading; if information about health consequences, also code 5.1, Information about health consequences, if about emotional consequences, also code 5.6, Information about emotional consequences; if about social, environmental or unspecified consequences also code 5.3, Information about social and environmental consequences | Present a speech given by a high status professional to emphasise the importance of not exposing patients to unnecessary radiation by ordering x-rays for back pain |
| **9.2** | Pros and cons | Advise the person to identify and compare reasons for wanting (pros) and not wanting to (cons) change the behavior (includes 'Decisional balance') Note: if providing information about health consequences, also code 5.1, Information about health consequences; if providing information about emotional consequences, also code 5.6, Information about emotional consequences; if providing information about social, environmental or unspecified consequences also code 5.3, Information about social and environmental consequences | Advise the person to list and compare the advantages and disadvantages of prescribing antibiotics for upper respiratory tract infections |
| **9.3** | Comparative imagining of future outcomes | Prompt or advise the imagining and comparing of future outcomes of changed versus unchanged behaviour | Prompt the person to imagine and compare likely or possible outcomes following attending versus not attending a screening appointment |
|  |  |  |  |
| **10** | **Reward and threat** | |  |
| **10.1** | Material incentive (behavior) | Inform that money, vouchers or other valued objects will be delivered if and only if there has been effort and/or progress in performing the behavior (includes 'Positive reinforcement') Note: if incentive is social, code 10.5, Social incentive if unspecified code 10.6, Non-specific incentive, and not 10.1, Material incentive (behavior); if incentive is for outcome, code 10.8, Incentive (outcome). If reward is delivered also code one of: 10.2, Material reward (behavior); 10.3, Non-specific reward; 10.4, Social reward, 10.9, Self-reward; 10.10, Reward (outcome) | Inform that a financial payment will be made each month in pregnancy that the woman has not smoked |
| **10.2** | Material reward (behavior) | Arrange for the delivery of money, vouchers or other valued objects if and only if there has been effort and/or progress in performing the behavior (includes 'Positive reinforcement') Note: If reward is social, code 10.4, Social reward, if unspecified code 10.3, Non-specific reward, and not 10.1, Material reward (behavior); if reward is for outcome, code 10.10, Reward (outcome). If informed of reward in advance of rewarded behaviour, also code one of: 10.1, Material incentive (behaviour); 10.5, Social incentive; 10.6, Non-specific incentive; 10.7, Self-incentive; 10.8, Incentive (outcome) | Arrange for the person to receive money that would have been spent on cigarettes if and only if the smoker has not smoked for one month |
| **10.3** | Non-specific reward | Arrange delivery of a reward if and only if there has been effort and/or progress in performing the behavior (includes 'Positive reinforcement') Note: if reward is material, code 10.2, Material reward (behavior), if social, code 10.4, Social reward, and not 10.3, Non-specific reward; if reward is for outcome code 10.10, Reward (outcome). If informed of reward in advance of rewarded behaviour, also code one of: 10.1, Material incentive (behaviour); 10.5, Social incentive; 10.6, Non-specific incentive; 10.7, Self-incentive; 10.8, Incentive (outcome) | Identify something (e.g. an activity such as a visit to the cinema) that the person values and arrange for this to be delivered if and only if they attend for health screening |
| **10.4** | Social reward | Arrange verbal or non-verbal reward if and only if there has been effort and/or progress in performing the behavior (includes 'Positive reinforcement')  Note: if reward is material, code 10.2, Material reward (behavior), if unspecified code 10.3, Non-specific reward, and not 10.4, Social reward; if reward is for outcome code 10.10, Reward (outcome). If informed of reward in advance of rewarded behaviour, also code one of: 10.1, Material incentive (behaviour); 10.5, Social incentive; 10.6, Non-specific incentive; 10.7, Self-incentive; 10.8, Incentive (outcome) | Congratulate the person for each day they eat a reduced fat diet |
| **10.5** | Social incentive | Inform that a verbal or non-verbal reward will be delivered if and only if there has been effort and/or progress in performing the behavior (includes 'Positive reinforcement')   Note: if incentive is material, code 10.1, Material incentive (behavior), if unspecified code 10.6, Non-specific incentive, and not 10.5, Social incentive; if incentive is for outcome code 10.8, Incentive (outcome). If reward is delivered also code one of: 10.2, Material reward (behavior); 10.3, Non-specific reward; 10.4, Social reward, 10.9, Self-reward; 10.10, Reward (outcome) | Inform that they will be congratulated for each day they eat a reduced fat diet |
| **10.6** | Non-specific incentive | Inform that a reward will be delivered if and only if there has been effort and/or progress in performing the behavior (includes 'Positive reinforcement')  Note: if incentive is material, code 10.1, Material incentive (behavior), if social, code 10.5, Social incentive and not 10.6, Non-specific incentive; if incentive is for outcome code 10.8, Incentive (outcome). If reward is delivered also code one of: 10.2, Material reward (behavior); 10.3, Non-specific reward; 10.4, Social reward, 10.9, Self-reward; 10.10, Reward (outcome) | Identify an activity that the person values and inform them that this will happen if and only if they attend for health screening |
| **10.7** | Self-incentive | Plan to reward self in future if and only if there has been effort and/or progress in performing the behavior  Note: if self-reward is material, also code 10.1, Material incentive (behavior), if social, also code 10.5, Social incentive, if unspecified, also code 10.6, Non-specific incentive; if incentive is for outcome code 10.8, Incentive (outcome). If reward is delivered also code one of: 10.2, Material reward (behavior); 10.3, Non-specific reward; 10.4, Social reward, 10.9, Self-reward; 10.10, Reward (outcome) | Encourage to provide self with material (e.g., new clothes) or other valued objects if and only if they have adhered to a healthy diet |
| **10.8** | Incentive (outcome) | Inform that  a reward will be delivered if and only if there has been effort and/or progress in achieving the behavioural outcome (includes 'Positive reinforcement')  Note: this includes social, material, self- and non-specific incentives for outcome; if incentive is for the behavior code 10.5, Social incentive, 10.1, Material incentive (behavior), 10.6, Non-specific incentive or 10.7, Self-incentive and not 10.8, Incentive (outcome). If reward is delivered also code one of: 10.2, Material reward (behavior); 10.3, Non-specific reward; 10.4, Social reward, 10.9, Self-reward; 10.10, Reward (outcome) | Inform the person that they will receive money if and only if a certain amount of weight is lost |
| **10.9** | Self-reward | Prompt self-praise or self-reward if and only if there has been effort and/or progress in performing the behavior  Note: if self-reward is material, also code 10.2, Material reward (behavior), if social, also code 10.4, Social reward, if unspecified, also code 10.3, Non-specific reward; if reward is for outcome code 10.10, Reward (outcome). If informed of reward in advance of rewarded behaviour, also code one of: 10.1, Material incentive (behaviour); 10.5, Social incentive; 10.6, Non-specific incentive; 10.7, Self-incentive; 10.8, Incentive (outcome) | Encourage to reward self with material (e.g., new clothes) or other valued objects if and only if they have adhered to a healthy diet |
| **10.10** | Reward (outcome) | Arrange for the delivery of a reward if and only if there has been effort and/or progress in achieving the behavioral outcome (includes 'Positive reinforcement')  Note: this includes social, material, self- and non-specific rewards for outcome; if reward is for the behavior code 10.4, Social reward, 10.2, Material reward (behavior), 10.3, Non-specific reward or 10.9, Self-reward and not 10.10, Reward (outcome). If informed of reward in advance of rewarded behaviour, also code one of: 10.1, Material incentive (behaviour); 10.5, Social incentive; 10.6, Non-specific incentive; 10.7, Self-incentive; 10.8, Incentive (outcome) | Arrange for the person to receive money if and only if a certain amount of weight is lost |
| **10.11** | Future punishment | Inform that future punishment or removal of reward will be a consequence of performance of an unwanted behavior (may include fear arousal) (includes 'Threat') | Inform that continuing to consume 30 units of alcohol per day is likely to result in loss of employment if the person continues |
|  |  |  |  |
| **11** | **Regulation** | |  |
| **11.1** | Pharmacological support | Provide, or encourage the use of or adherence to, drugs to facilitate behavior change   Note: if pharmacological support to reduce negative emotions (i.e. anxiety) then also code 11.2, Reduce negative emotions | Suggest the patient asks the family physician for nicotine replacement therapy to facilitate smoking cessation |
| **11.2** | Reduce negative emotions | Advise on ways of reducing negative emotions to facilitate performance of the behavior (includes 'Stress Management')  Note: if includes analysing the behavioural problem, also code 1.2, Problem solving | Advise on the use of stress management skills, e.g. to reduce anxiety about joining Alcoholics Anonymous |
| **11.3** | Conserving mental resources | Advise on ways of minimising demands on mental resources to facilitate behavior change | Advise to carry food calorie content information to reduce the burden on memory in making food choices |
| **11.4** | Paradoxical instructions | Advise to engage in some form of the unwanted behavior with the aim of reducing motivation to engage in that behaviour | Advise a smoker to smoke twice as many cigarettes a day as they usually do Tell the person to stay awake as long as possible in order to reduce insomnia |
| **12** | **Antecedents** | |  |
| **12.1** | Restructuring the physical environment | Change, or advise to change the physical environment in order to facilitate performance of the wanted behavior or create barriers to the unwanted behavior (other than prompts/cues, rewards and punishments)  Note: this may also involve 12.3, Avoidance/reducing exposure to cues for the behavior; if restructuring of the social environment code 12.2, Restructuring the social environment; if only adding objects to the environment, code 12.5, Adding objects to the environment | Advise to keep biscuits and snacks in a cupboard that is inconvenient to get to Arrange to move vending machine out of the school |
| **12.2** | Restructuring the social environment | Change, or advise to change the social environment in order to facilitate performance of the wanted behavior or create barriers to the unwanted behavior (other than prompts/cues, rewards and punishments)    Note: this may also involve 12.3, Avoidance/reducing exposure to cues for the behavior; if also restructuring of the physical environment also code 12.1, Restructuring the physical environment | Advise to minimise time spent with friends who drink heavily to reduce alcohol consumption |
| **12.3** | Avoidance/reducing exposure to cues for the behavior | Advise on how to avoid exposure to specific social and contextual/physical cues for the behavior, including changing daily or weekly routines  Note: this may also involve 12.1, Restructuring the physical environment and/or 12.2, Restructuring the social environment; if the BCT includes analysing the behavioral problem, only code 1.2, Problem solving | Suggest to a person who wants to quit smoking that their social life focus on activities other than pubs and bars which have been associated with smoking |
| **12.4** | Distraction | Advise or arrange to use an alternative focus for attention to avoid triggers for unwanted behaviour | Suggest to a person who is trying to avoid between-meal snacking to focus on a topic they enjoy (e.g. holiday plans) instead of focusing on food |
| **12.5** | Adding objects to the environment | Add objects to the environment in order to facilitate performance of the behavior  Note: Provision of information (e.g. written, verbal, visual) in a booklet or leaflet is insufficient. If this is accompanied by social support, also code 3.2, Social support (practical); if the environment is changed beyond the addition of objects, also code 12.1, Restructuring the physical environment | Provide free condoms to facilitate safe sex Provide attractive toothbrush to improve tooth brushing technique |
| **12.6** | Body changes | Alter body structure, functioning or support directly to facilitate behavior change | Prompt strength training, relaxation training or provide assistive aids (e.g. a hearing aid) |
|  |  |  |  |
| **13** | **Identity** |  |  |
| **13.1** | Identification of self as role model | Inform that one's own behavior may be an example to others | Inform the person that if they eat healthily, that may be a good example for their children |
| **13.2** | Framing/  reframing | Suggest the deliberate adoption of a perspective or new perspective on behavior (e.g. its purpose) in order to change cognitions or emotions about performing the behavior (includes 'Cognitive structuring'); If information about consequences then code 5.1, Information about health consequences, 5.6, Information about emotional consequences or 5.3, Information about social and environmental consequences instead of 13.2, Framing/reframing | Suggest that the person might think of the tasks as reducing sedentary behavior (rather than increasing activity) |
| **13.3** | Incompatible beliefs | Draw attention to discrepancies between current or past behavior and self-image, in order to create discomfort (includes 'Cognitive dissonance') | Draw attention to a doctor's liberal use of blood transfusion and their self-identification as a proponent of evidence-based medical practice |
| **13.4** | Valued self-identity | Advise the person to write or complete rating scales about a cherished value or personal strength as a means of affirming the person's identity as part of a behavior change strategy  (includes 'Self-affirmation') | Advise the person to write about their personal strengths before they receive a message advocating the behavior change |
| **13.5** | Identity associated with changed behavior | Advise the person to construct a new self-identity as someone who 'used to engage with the unwanted behavior' | Ask the person to articulate their new identity as an 'ex-smoker' |
|  |  |  |  |
| **14** | **Scheduled consequences** | |  |
| **14.1** | Behavior cost | Arrange for withdrawal of something valued if and only if an unwanted behavior is performed (includes 'Response cost'). Note if withdrawal of contingent reward code, 14.3, Remove reward | Subtract money from a prepaid refundable deposit when a cigarette is smoked |
| **14.2** | Punishment | Arrange for aversive consequence contingent on the performance of the unwanted behavior | Arrange for the person to wear unattractive clothes following consumption of fatty foods |
| **14.3** | Remove reward | Arrange for discontinuation of  contingent reward following performance of the unwanted behavior (includes 'Extinction') | Arrange for the other people in the household to ignore the person every time they eat chocolate (rather than attending to them by criticising or persuading) |
| **14.4** | Reward approximation | Arrange for reward following any approximation to the target behavior, gradually rewarding only performance closer to the wanted behavior (includes 'Shaping')  Note: also code one of 59-63 | Arrange reward for any reduction in daily calories, gradually requiring the daily calorie count to become closer to the planned calorie intake |
| **14.5** | Rewarding completion | Build up behavior by arranging reward following final component of the behavior; gradually add the components of the behavior that occur earlier in the behavioral sequence (includes 'Backward chaining')  Note: also code one of 10.2, Material reward (behavior); 10.3, Non-specific reward; 10.4, Social reward, 10.9, Self-reward; 10.10, Reward (outcome) | Reward eating a supplied low calorie meal; then make reward contingent on cooking and eating the meal; then make reward contingent on purchasing, cooking and eating the meal |
| **14.6** | Situation-specific reward | Arrange for reward following the behavior in one situation but not in another (includes 'Discrimination training')   Note: also code one of 10.2, Material reward (behavior); 10.3, Non-specific reward; 10.4, Social reward, 10.9, Self-reward; 10.10, Reward (outcome) | Arrange reward for eating at mealtimes but not between meals |
| **14.7** | Reward incompatible behavior | Arrange reward for responding in a manner that is incompatible with a previous response to that situation (includes 'Counter-conditioning')  Note: also code one of 10.2, Material reward (behavior); 10.3, Non-specific reward; 10.4, Social reward, 10.9, Self-reward; 10.10, Reward (outcome)  Arrange reward for ordering a soft drink at the bar rather than an alcoholic beverage | Arrange reward  for ordering a soft drink at the bar rather than an alcoholic beverage |
| **14.8** | Reward alternative behavior | Arrange reward for performance of an alternative to the unwanted behavior (includes 'Differential reinforcement')  Note: also code one of 10.2, Material reward (behavior); 10.3, Non-specific reward; 10.4, Social reward, 10.9, Self-reward; 10.10, Reward (outcome); consider also coding 1.2, Problem solving | Reward for consumption of low fat foods but not consumption of high fat foods |
| **14.9** | Reduce reward frequency | Arrange for rewards to be made contingent on increasing duration or frequency of the behavior (includes 'Thinning')  Note: also code one of 10.2, Material reward (behavior); 10.3, Non-specific reward; 10.4, Social reward, 10.9, Self-reward; 10.10, Reward (outcome) | Arrange reward for each day without smoking, then each week, then each month, then every 2 months and so on |
| **14.10** | Remove punishment | Arrange for removal of an unpleasant consequence contingent on performance of the wanted behavior (includes 'Negative reinforcement') | Arrange for someone else to do housecleaning only if the person has adhered to the medication regimen for a week |
|  |  |  |  |
| **15** | **Self-belief** |  |  |
| **15.1** | Verbal persuasion about capability | Tell the person that they can successfully perform the wanted behavior, arguing against self-doubts and asserting that they can and will succeed | Tell the person that they can successfully increase their physical activity, despite their recent heart attack. |
| **15.2** | Mental rehearsal of successful performance | Advise to practise imagining performing the behavior successfully in relevant contexts | Advise to imagine eating and enjoying a salad in a work canteen |
| **15.3** | Focus on past success | Advise to think about or list previous successes in performing the behavior (or parts of it) | Advise to describe or list the occasions on which the person had ordered a non-alcoholic drink in a bar |
| **15.4** | Self-talk | Prompt positive self-talk (aloud or silently) before and during the behavior | Prompt the person to tell themselves that a walk will be energising |
|  |  |  |  |
| **16** | **Covert learning** | |  |
| **16.1** | Imaginary punishment | Advise to imagine performing the unwanted behavior in a real-life situation followed by imagining an unpleasant consequence (includes 'Covert sensitisation') | Advise to imagine overeating and then vomiting |
| **16.2** | Imaginary reward | Advise to imagine performing the wanted behavior in a real-life situation followed by imagining a pleasant consequence (includes 'Covert conditioning') | Advise the health professional to imagine giving dietary advice followed by the patient losing weight and no longer being diabetic |
| **16.3** | Vicarious consequences | Prompt observation of the consequences (including rewards and punishments) for others when they perform the behavior | Draw attention to the positive comments other staff get when they disinfect their hands regularly |
| **Delivery techniques** | | | |
| Information delivery | | Typically involves one-way interactions from the system to the user upon access. | |
| Notifications | | Individual messages pushed to the user, such as text messages, emails, or in-app notifications. | |
| Logs | | A form of data collection that require the user to enter data. Examples include free entry, selection menus, and using a rating scale. | |
| Passive data collection | | Data collected without any user effort, such as phone sensor data collection and data from external devices such as wearables/smartwatches. | |
| Messaging | | Forms include one-way or bidirectional interactions with the intervention lead, discussion boards with other participants as a social support tool, or notifications to aid compliance (e.g., food log reminders). | |
| Reports | | Reflections of data collected provided back to the user (e.g., food records or calorie counts). | |
| Gamification | | The application of common game elements in a non-game setting, often used as an online marketing technique to encourage engagement with a product or service. Includes point scoring and competitions with peers. | |
| Media | | Refers to content delivered in text, video, or audio format. The medium used can differ for various elements of the intervention to make them more engaging and/or informative for the user. | |
| Personalisation | | Tailoring the intervention using predetermined/self-set criteria or machine learning methods to automatically adapt the intervention content based on the user’s previous behaviour and/or context. Examples of personalised elements include goal-setting, action plans, and feedback on progress towards goals. | |

## **Table 7. Reporting transparency and risk of bias checklist.**

| Item number | Item | Source |
| --- | --- | --- |
| *Aim* | | |
| 1 | Clearly stated research aim, question, or hypothesis. | CONSORT (adapted) |
| *Methods* | | |
| 2 | Appropriate measures were taken to reduce selection bias.  Methods to minimise selection bias include targeting community groups and low-income settings, while self-selection and volunteering techniques are more prone to bias. | CONSORT (adapted) |
| 3 | Reasons for non-response described. | Own item |
| 4 | How sample size was selected. | CONSORT |
| 5 | Comparable study groups on diet and key demographics.  Nb: Item not included for pre-post trials. | CONSORT (adapted) |
| 6 | Losses and exclusions after study commencement, together with reasons. | RoB2 (adapted) |
| 7 | Lost to FU missing at random: MAR/NMAR.  Nb: Studies were coded as NMAR (score = 1) if the study documented quantitative differences between the sample who dropped out and those who completed, whereas studies were coded as MAR (score = 0) when the samples were comparable. If the drop-out rate was 0%, studies were coded as NMAR. | Own item |
| 8 | Adequate randomisation method. | RoB2 |
| 9 | Outcome assessors sufficiently blinded.  Nb: Item not included for pre-post trials. | RoB2 |
| 10 | Participants sufficiently blinded.  Nb: Item not included for pre-post trials. | RoB2 |
| 11 | Both outcome assessors and participants sufficiently blinded (double blind).  Nb: Item not included for pre-post trials. | RoB2 |
| *Intervention* | | |
| 12 | Intervention described in detail in terms of theory/rationale behind the intervention. | TidIER |
| 13 | Intervention described in detail in terms of behaviour change techniques. | TidIER |
| 14 | Intervention described in detail in terms of delivery techniques. | TidIER |
| 15 | Intervention described in detail in terms of theory, behaviour change techniques and delivery techniques. | TidIER |
| 16 | Completely defined pre-specified primary and secondary outcome measures, including how and when they were assessed. | RoB2 |
| 17 | Dietary measurements valid and reliable. | CONSORT (adapted) |
| 18 | Appropriate statistical analysis. | CONSORT |
| 19 | Appropriate methods for additional analyses, such as subgroup analyses and adjusted analyses.  Nb: A score of 0 was given to studies without mention of additional analyses. | RoB2 |
| *Results* | | |
| 20 | A table showing baseline demographic and clinical characteristics for each group. | RoB2 |
| 21 | For each primary and secondary outcome, results for each group in means, SDs and Ns (including non-sig results), and the estimated effect size and its precision (such as 95% confidence interval) if applicable. | RoB2 (adapted) |
| 22 | All important harms or unintended effects in each group. | RoB2 |
| *Discussion* | | |
| 23 | Conclusions supported by results with consideration for biases and limitations. | CONSORT |
| *Registration and funding* | | |
| 24 | Registration number and name of trial registry. | RoB2 |
| 25 | Where the full trial protocol can be accessed, if available. | RoB2 |
| 26 | Funding bias unlikely, i.e., funded by an academic or research institution, not by industry (adapted). | CONSORT (adapted) |

## **Table 8. Portion size estimates.**

| Food group | Size (g) | Reference(s) |
| --- | --- | --- |
| Fruit and vegetables | 90 | - USA: 1 cup cut-up fruit or one medium whole fruit^9^ - European Commission (EU): 80-100g^4^ - UK: 80g^10^ |
| Legumes | 150 | - UK: 150g as a protein source not a vegetable portion^10^ - Australia: 150g as a protein source not a vegetable portion^11^ |
| Dairy | 200 | - UK: 200ml milk^10^ - Spain: 200-250ml milk^12^ |
| Meat | 90 | - UK: 90g cooked meat (beef, pork, lamb, mince, chicken, or turkey)^10^ - Australia: 90-100g raw red meat or 100g raw poultry^13^ |

## **Table 9. List of excluded studies with reasons.**

| **Title** | **Authors** | **Published Year** | **Journal/Source** | **Volume** | **Issue** | **Pages** | **Reason for exclusion** |
| --- | --- | --- | --- | --- | --- | --- | --- |
| Xhibition kitchen northeastern dining enhancing experiential learning in higher education | Timmons, M.; Fantasia, D.; Barton, T. | 2018 | Journal of Alternative and Complementary Medicine | 24 | 7 | A17 | Wrong intervention |
| Weight reduction through a digital nutrition and food purchasing platform among users with obesity: Longitudinal study | Hu, E. A.; Nguyen, V.; Langheier, J.; Shurney, D. | 2020 | Journal of Medical Internet Research | 22 | 9 | e19634 | Wrong intervention |
| Weight loss support for nurses: Apps and healthy food options: Resources designed to support healthcare workers' well-being that can help you lose weight and change how you think about food | Trueland, Jennifer | 2022 | Nursing Standard | 37 | 8 | 70-73 | Wrong intervention |
| Using new technologies to promote weight management: a randomised controlled trial study protocol | Jane, M.; Foster, J.; Hagger, M.; Pal, S. | 2015 | BMC Public Health | 15 | 509 |  | Wrong intervention |
| Use of mobile health tools in controlling overweight and eating habits: results of a Russian multicenter randomized trial | Kulikova, M. S.; Kalinina, A. M.; Eganyan, R. A.; Kontsevaya, A. V.; Drapkina, O. M. | 2022 | Profilakticheskaya Meditsina | 25 | 12 | 46-54 | Wrong intervention |
| Use of a computerized tracking system to monitor and provide feedback on dietary goals for calorie-restricted diets: The POUNDS LOST study | Anton, S. D.; LeBlanc, E.; Allen, H. R.; Karabetian, C.; Sacks, F.; Bray, G.; Williamson, D. A. | 2012 | Journal of Diabetes Science and Technology | 6 | 5 | 1216-1225 | Wrong intervention |
| Use a web-app to improve breast cancer risk factors and symptoms knowledge and adherence to healthy diet and physical activity in women without breast cancer diagnosis (Precam project) | Martin-Payo, Ruben; Martinez-Urquijo, Andrea; Zabaleta-Del-Olmo, Edurne; Del Mar Fernandez-Alvarez, Maria | 2023 | Cancer causes & control : CCC | 34 | 2 | 113-122 | Wrong intervention |
| The impact of an m-Health financial incentives program on the physical activity and diet of Australian truck drivers | Gilson, N. D.; Pavey, T. G.; Wright, O. R. L.; Vandelanotte, C.; Duncan, M. J.; Gomersall, S.; Trost, S. G.; Brown, W. J. | 2017 | BMC Public Health | 17 | 467 |  | Wrong intervention |
| The impact of a Web-based app (eBalance) in promoting healthy lifestyles: Randomized controlled trial | Safran Naimark, Jenny; Madar, Zecharia; Shahar, Danit R. | 2015 | Journal of Medical Internet Research | 17 | 3 | No-Specified | Wrong intervention |
| The impact of a multilevel childhood obesity prevention intervention on healthful food acquisition, preparation, and fruit and vegetable consumption on African-American adult caregivers | Trude, Angela C. B.; Surkan, Pamela J.; Anderson Steeves, Elizabeth; Pollack Porter, Keshia; Gittelsohn, Joel | 2019 | Public Health Nutrition | 22 | 7 | 1300-1315 | Wrong intervention |
| Sustaining weight loss among adults with obesity using a digital meal planning and food purchasing platform for 12, 24, and 36 months: a longitudinal study | Hu, E. A.; Pasupuleti, M.; Nguyen, V.; Langheier, J.; Shurney, D. | 2021 | Nutrition Journal | 20 | 1 | 8 | Wrong intervention |
| Smartphone technology and text messaging for weight loss in young adults: A randomized controlled trial | Stephens, Janna D.; Yager, Allison M.; Allen, Jerilyn | 2017 | Journal of Cardiovascular Nursing | 32 | 1 | 39-46 | Wrong intervention |
| Real-world effectiveness of digital and group-based lifestyle interventions as compared with usual care to reduce type 2 diabetes risk - A stop diabetes pragmatic randomised trial | Lakka, Timo A.; Aittola, Kirsikka; Jarvela-Reijonen, Elina; Tilles-Tirkkonen, Tanja; Mannikko, Reija; Lintu, Niina; Karhunen, Leila; Kolehmainen, Marjukka; Harjumaa, Marja; Mattila, Elina; Jarvenpaa, Riia; Ermes, Miikka; Mikkonen, Santtu; Martikainen, Janne; Poutanen, Kaisa; Schwab, Ursula; Absetz, Pilvikki; Lindstrom, Jaana; Pihlajamaki, Jussi | 2023 | The Lancet regional health. Europe | 24 |  | 100527 | Wrong intervention |
| Randomized test of an implementation intention-based tool to reduce stress-induced eating | O'Connor, Daryl B.; Armitage, Christopher J.; Ferguson, Eamonn | 2015 | Annals of Behavioral Medicine | 49 | 3 | 331-343 | Wrong intervention |
| Online self-tracking groups to increase fruit and vegetable intake: A small-scale study on mechanisms of group effect on behavior change | Meng, Jingbo; Peng, Wei; Shin, Soo Yun; Chung, Minwoong | 2017 | Journal of Medical Internet Research | 19 | 3 | 159-173 | Wrong intervention |
| Nourish | Kadey, Matthew | 2021 | IDEA Fitness Journal | |  | 37-43 | Wrong intervention |
| Multiple behavior changes in diet and activity: A randomized controlled trial using mobile technology | Spring, B.; Schneider, K.; McFadden, H. G.; Vaughn, J.; Kozak, A. T.; Smith, M.; Moller, A. C.; Epstein, L. H.; DeMott, A.; Hedeker, D.; Siddique, J.; Lloyd-Jones, D. M. | 2012 | Archives of Internal Medicine | 172 | 10 | 789-796 | Wrong intervention |
| Multicomponent mHealth Intervention for Large, Sustained Change in Multiple Diet and Activity Risk Behaviors: The Make Better Choices 2 Randomized Controlled Trial | Spring, Bonnie; Pellegrini, Christine; McFadden, H. G.; Pfammatter, Angela Fidler; Stump, Tammy K.; Siddique, Juned; King, Abby C.; Hedeker, Donald | 2018 | Journal of Medical Internet Research | 20 | 6 | 01-Jan | Wrong intervention |
| Mobile tablet menus: attractiveness and impact of nutrition labeling formats on millennials' food choices | Yepes, M. F. | 2015 | Cornell Hospitality Quarterly | 56 | 1 | 58-67 | Wrong intervention |
| Mindful decision making and inhibitory control training as complementary means to decrease snack consumption | Forman, E. M.; Shaw, J. A.; Goldstein, S. P.; Butryn, M. L.; Martin, L. M.; Meiran, N.; Crosby, R. D.; Manasse, S. M. | 2016 | Appetite | 103 |  | 176-183 | Wrong intervention |
| Mass dissemination of web and smartphone-delivered food response inhibition training to reduce unhealthy snacking | Lawrence, N. S.; Van Beurden, S.; Javaid, M.; Mostazir, M. M. | 2018 | Appetite | 130 |  | 309 | Wrong intervention |
| Intermittent fasting, Paleolithic, or Mediterranean diets in the real world: exploratory secondary analyses of a weight-loss trial that included choice of diet and exercise | Jospe, Michelle R.; Roy, Melyssa; Brown, Rachel C.; Haszard, Jillian J.; Meredith-Jones, Kim; Fangupo, Louise J.; Osborne, Hamish; Fleming, Elizabeth A.; Taylor, Rachael W. | 2020 | American Journal of Clinical Nutrition | 111 | 3 | 503-514 | Wrong intervention |
| Instagram-based priming to nudge drink choices: Subtlety is not the answer | Kay, E.; Kemps, E.; Prichard, I.; Tiggemann, M. | 2023 | Appetite | 180 |  | 106337 | Wrong intervention |
| How eating-related social media postings influence healthy eating in senders and network members: Two field experiments with intensive longitudinal data | Kilb, M.; Giese, H.; Mata, J. | 2023 | Appetite | 182 |  | 106430 | Wrong intervention |
| Healthy Children, Strong Families 2: a randomized controlled trial of a healthy lifestyle intervention for American Indian families designed using community-based approaches | Tomayko, E. J.; Prince, R. J.; Cronin, K. A.; Parker, T.; Kim, KyungMann; Grant, V. M.; Sheche, J. N.; Adams, A. K. | 2017 | Clinical Trials | 14 | 2 | 152-161 | Wrong intervention |
| HEALTH (Healthy Eating, Activity, Lifestyle Training Headquarters) internet/mobile weight management program for the U.S. Army: Outcomes and future directions | Stewart, T.; Beyl, R.; Switzer, M.; Friedl, K.; Young, A.; Ryan, D.; Williamson, D. | 2017 | Journal of Science and Medicine in Sport | 20 |  | S34-S35 | Wrong intervention |
| Effects of the e-motivate4change program on metabolic syndrome in young adults using health apps and wearable devices: Quasi-experimental study | Lee, J. S.; Kang, M. A.; Lee, S. K. | 2020 | Journal of Medical Internet Research | 22 | 7 | e17031 | Wrong intervention |
| Effectiveness of Web-Based Personalized Nutrition Advice for Adults Using the eNutri Web App: Evidence From the EatWellUK Randomized Controlled Trial | Zenun Franco, Rodrigo; Fallaize, Rosalind; Weech, Michelle; Hwang, Faustina; Lovegrove, Julie A. | 2022 | Journal of medical Internet research | 24 | 4 | e29088 | Wrong intervention |
| Effectiveness of an mHealth Intervention Combining a Smartphone App and Smart Band on Body Composition in an Overweight and Obese Population: Randomized Controlled Trial (EVIDENT 3 Study) | Lugones-Sanchez, Cristina; Sanchez-Calavera, Maria Antonia; Repiso-Gento, Irene; Adalia, Esther G.; Ramirez-Manent, J. Ignacio; Agudo-Conde, Cristina; Rodriguez-Sanchez, Emiliano; Gomez-Marcos, Manuel Angel; Recio-Rodriguez, Jose I.; Garcia-Ortiz, Luis; Investigators, Evident | 2020 | JMIR mHealth and uHealth | 8 | 11 | e21771 | Wrong intervention |
| Effectiveness of a Weight Management Program Applying Mobile Health Technology as a Supporting Tool for Overweight and Obese Working Women | Siriwoen, Roscharin; Chongsuwat, Rewadee; Tansakul, Supreya; Siri, Sukhontha | 2018 | Asia-Pacific Journal of Public Health | 30 | 6 | 572-581 | Wrong intervention |
| Effectiveness of a Web- and mobile phone-based intervention to promote physical activity and healthy eating in middle-aged males: Randomized controlled trial of the ManUp study | Duncan, Mitch; Vandelanotte, Corneel; Kolt, Gregory S.; Rosenkranz, Richard R.; Caperchione, Cristina M.; George, Emma S.; Ding, Hang; Hooker, Cindy; Karunanithi, Mohan; Maeder, Anthony J.; Noakes, Manny; Tague, Rhys; Taylor, Pennie; Viljoen, Pierre; Mummery, W. Kerry | 2014 | Journal of Medical Internet Research | 16 | 6 | 40-60 | Wrong intervention |
| Design and implementation of a culturally tailored diet and lifestyle intervention for African and Caribbean people residing in Manchester: Insights from a process evaluation | Osei-Kwasi, H.; Akparibo, R.; Ojwang, A.; Asamane, E.; Olayanju, A.; Ellahi, B. | 2022 | Proceedings of the Nutrition Society | 81 |  | E226 | Wrong intervention |
| Changing diet and physical activity in nurses: a pilot study and process evaluation highlighting challenges in workplace health promotion | Torquati, L.; Kolbe-Alexander, T.; Pavey, T.; Leveritt, M. | 2018 | Journal of Nutrition Education and Behavior | 50 | 10 | 1015-1025 | Wrong intervention |
| 'Change4Life Smart Swaps': quasi-experimental evaluation of a natural experiment | Wrieden, W. L.; Levy, L. B. | 2016 | Public Health Nutrition | 19 | 13 | 2388-2392 | Wrong intervention |
| Assessment of cooking matters facebook platform to promote healthy eating behaviors among low-income caregivers of young children in the united states: A pilot study | Zhang, Q.; Panichelli, J.; Hall, L. A. | 2021 | Nutrients | 13 | 8 | 2694 | Wrong intervention |
| Assessing the effectiveness of a 4-week online intervention on food literacy and fruit and vegetable consumption in Australian adults: the online MedDiet challenge | Ng, A. H.; Elghattis, Y.; Biesiekierski, J. R.; Moschonis, G. | 2022 | Health and Social Care in the Community | 30 | 6 | e4975-e4981 | Wrong intervention |
| An ecological momentary episodic future thinking intervention on mother's weekly food purchases | Hollis-Hansen, Kelseanna; Seidman, Jennifer; O'Donnell, Sara; Wedderburn, Amber; Stanar, Sanja; Brande, Spencer; Epstein, Leonard H. | 2020 | Health Psychology | 39 | 2 | 159-167 | Wrong intervention |
| A Mobile Health Lifestyle Program for Prevention of Weight Gain in Young Adults (TXT2BFiT): Nine-Month Outcomes of a Randomized Controlled Trial | Allman-Farinelli, Margaret; Partridge, Stephanie Ruth; McGeechan, Kevin; Balestracci, Kate; Hebden, Lana; Wong, Annette; Phongsavan, Philayrath; Denney-Wilson, Elizabeth; Harris, Mark F.; Bauman, Adrian | 2016 | JMIR mHealth and uHealth | 4 | 2 | e78 | Wrong intervention |
| 8700 kJ-a community education campaign to support kilojoule labelling | Vineburg, J.; Moroney, C.; Kitchener, S.; Szabo, L.; Jansson, E.; Mitchell, J.; Eden, B.; Caterson, I. | 2013 | Obesity Facts | 6 |  | 9 | Wrong intervention |
| A randomized controlled trial on the efficacy of dietary interventions for improving the diet in government office workers in the Galle district | SLCTR/2020/025 | 2020 | https://trialsearch.who.int/Trial2.aspx?TrialID=SLCTR/2020/025 | | | | Wrong intervention |
| Online vs. Face-to-face Nutritional Advice in Kuwait (EatWellQ8) | NCT03396263 | 2018 | https://clinicaltrials.gov/show/NCT03396263 | | | | Wrong intervention |
| Promoting Lifestyle Change Via Tailored mHealth Feedback to Improve Health | NCT03367936 | 2017 | https://clinicaltrials.gov/show/NCT03367936 | | | | Wrong intervention |
| Episodic future thinking reduces eating in a food court | O'Neill, J; Daniel, TO; Epstein, LH | 2016 | Eating behaviors | 20 |  | Sep-13 | Wrong intervention |
| Use of Wearable Technology and Social Media to Improve Physical Activity and Dietary Behaviors among College Students: a 12-Week Randomized Pilot Study | Pope, ZC; Barr-Anderson, DJ; Lewis, BA; Pereira, MA; Gao, Z | 2019 | International journal of environmental research and public health | 16 | 19 |  | Wrong intervention |
| OL@-OR@ trial: a cluster-randomised controlled trial to evaluate a co-designed, culturally-tailored, lifestyle-support mobile health (mHealth) tool, which includes an app and website for Maori and Pasifika in New Zealand | ACTRN12617001484336 | 2017 | https://trialsearch.who.int/Trial2.aspx?TrialID=ACTRN12617001484336 | | | | Wrong intervention |
| Feasibility and preliminary efficacy of the 'HEYMAN' healthy lifestyle program for young men: a pilot randomised controlled trial | Ashton, L. M.; Morgan, P. J.; Hutchesson, M. J.; Rollo, M. E.; Collins, C. E. | 2017 | Nutrition Journal | 16 | 2 |  | Wrong intervention |
| A Contactless App-Based Intervention to Improve Health Behaviors in Airline Pilots: A Randomized Trial | Wilson, D.; Driller, M. W.; Johnston, B.; Gill, N. D. | 2023 | American Journal of Preventive Medicine | | | | Wrong intervention |
| A pilot-RCT of an app-based intervention to reduce the cardiovascular disease risk in shift-workers through targeting multiple lifestyle behaviours | ACTRN12618001785291 | 2018 | https://trialsearch.who.int/Trial2.aspx?TrialID=ACTRN12618001785291 | | | | Wrong intervention |
| Tweeting for nutrition: feasibility and efficacy outcomes of a 6-week social media-based nutrition education intervention for student-athletes. | Coccia C, Fernandes SM, Altiti J. | 2020 | The Journal of Strength & Conditioning Research | 34 | 7 | 2084–92 | Wrong intervention |
| Mobile application-based dietary sugar intake reduction intervention study according to the stages of behavior change in female college students | Choi, YunJung; Kim, HyunSook | 2019 | Journal of Nutrition and Health | 52 | 5 | 488-500 | Wrong language |
| Using a smartphone app in changing cardiovascular risk factors: A randomized controlled trial (EVIDENT II study) | Gonzalez-Sanchez, J.; Recio-Rodriguez, J. I.; Fernandez-delRio, A.; Sanchez-Perez, A.; Magdalena-Belio, J. F.; Gomez-Marcos, M. A.; Garcia-Ortiz, L.; Rodriguez-Sanchez, E.; Maderuelo-Fernandez, J. A.; Iglesias-Valiente, J. A.; Patino-Alonso, M. C.; Perez-Arechaederra, D.; Mora-Simon, S.; Agudo-Conde, C.; Castano-Sanchez, M. C.; Rodriguez-Martin, C.; Sanchez-Salgado, B.; de Cabo-Laso, A.; Alonso-Dominguez, R.; Sanchez-Aguadero, N.; Martin-Cantera, C.; Canales-Reina, J.; de Pablo, E. R.; Lasaosa-Medina, M. L.; Calvo-Aponte, M. J.; Rodriguez-Franco, A.; Martin-Borras, C.; Puig-Ribera, A.; Colominas-Garrido, R.; Puig, E. D.; Romaguera-Bosch, M.; Maneus, S.; Schmolling-Guinovart, Y.; Rodriguez-Martin, B.; del Rio, A. F.; Fernandez-Diaz, J. A.; Calderon-Ubeda, J. B.; Menendez-Obregon, J. L.; Segura-Fragoso, A.; Zabala-Banos, C.; Martinez-Vizcaino, V.; Martinez-Andres, M.; Fernandez-Alonso, M. C.; Gamez-Arranz, A.; de la Fuente, A. D.; Menendez-Suarez, M.; Repiso-Gento, I.; Arranz-Hernando, M. I.; Perez-Concejo, M. I.; Alonso-Manjarres, M. A.; Villarroya, M. E.; de Rodrigo, M. J. A.; de Lis, M. P.; de Arriba-Gomez, M. D.; Arqueaga-Luengo, A.; Lopez-Arroyo, M. M.; Gonzalez-Viejo, N.; Otegui-Ilarduya, L.; Rubio-Galan, F. J.; Melguizo-Bejar, A.; Sauras-Yera, I.; Gil-Train, M. J.; Iribarne-Ferrer, M.; Magdalena-Gonzalez, O.; Lafuente-Ripolles, M. A.; Grandes, G.; Sanchez, A.; Arce, V.; Arietaleanizbeaskoa, M. S.; Mendizabal, N.; Iturregui-San Nicolas, E.; Grp, Evident Investigators | 2019 | INTERNATIONAL JOURNAL OF MEDICAL INFORMATICS | 125 |  | 13-21 | Wrong outcome |
| Tweeting to Health: a novel mHealth intervention using Fitbits and Twitter to foster healthy lifestyles | Chung, A. E.; Skinner, A. C.; Hasty, S. E.; Perrin, E. M. | 2017 | Clinical Pediatrics | 56 | 1 | 26-32 | Wrong outcome |
| The lifana solution: a mobile health personalized nutrition application for promoting healthy diet in elderly people | Ferrini, K.; Stahl, C.; Bohn, T. | 2020 | Clinical Nutrition ESPEN | 40 |  | 586 | Wrong outcome |
| Supporting Healthy Grocery Shopping via Mobile Augmented Reality | Ahn, J.; Williamson, J.; Gartrell, M.; Han, R.; Lv, Q.; Mishra, S. | 2015 | ACM TRANSACTIONS ON MULTIMEDIA COMPUTING COMMUNICATIONS AND APPLICATIONS | 12 | 1 |  | Wrong outcome |
| Randomized controlled trial of OnTrack, a just-in-time adaptive intervention designed to enhance weight loss | Forman, E. M.; Goldstein, S. P.; Crochiere, R. J.; Butryn, M. L.; Juarascio, A. S.; Zhang, F.; Foster, G. D. | 2019 | Translational Behavioral Medicine | 9 | 6 | 989-1001 | Wrong outcome |
| Outcomes in a digital weight management intervention with one-on-one health coaching | Silberman, J. M.; Kaur, M.; Sletteland, J.; Venkatesan, A. | 2020 | PLoS ONE | 15 | 4 | e0232221 | Wrong outcome |
| Nudging for eco-friendly online shopping-Attraction effect curbs price sensitivity | Guath, Mona; Stikvoort, Britt; Juslin, Peter | 2022 | Journal of Environmental Psychology | 81 |  | 01-Nov | Wrong outcome |
| Mobile Phone App for Self-Monitoring of Eating Rhythm: Field Experiment | Pentikainen, Saara; Tanner, Hannu; Karhunen, Leila; Kolehmainen, Marjukka; Poutanen, Kaisa; Pennanen, Kyosti | 2019 | JMIR mHealth and uHealth | 7 | 3 | e11490 | Wrong outcome |
| Investigating the feasibility and acceptability of using Instagram to engage post-graduate students in a mass communication social media-based health intervention, #WeeStepsToHealth | O'Kane, Niamh; McKinley, Michelle C.; Gough, Aisling; Hunter, Ruth F. | 2022 | Pilot and feasibility studies | 8 | 1 | 254 | Wrong outcome |
| Insights on the Effect and Experience of a Diet-Tracking Application for Older Adults in a Diet Trial | van der Lubbe, L. M.; Klein, M. C. A.; Visser, M.; Wijnhoven, H. A. H.; Reinders, I. | 2022 | TECHNOLOGIES | 10 | 1 |  | Wrong outcome |
| Efficacy of the Mindfulness Meditation Mobile App "Calm" to Reduce Stress Among College Students: Randomized Controlled Trial | Huberty, Jennifer; Green, Jeni; Glissmann, Christine; Larkey, Linda; Puzia, Megan; Lee, Chong | 2019 | JMIR mHealth and uHealth | 7 | 6 | e14273 | Wrong outcome |
| Effectiveness of App-Based Intervention to Improve Health Status of Sedentary Middle-Aged Males and Females | Martinez-Olcina, M.; Cuestas-Calero, B. J.; Miralles-Amoros, L.; Vicente-Martinez, M.; Sanchez-Sanchez, J. | 2022 | INTERNATIONAL JOURNAL OF ENVIRONMENTAL RESEARCH AND PUBLIC HEALTH | 19 | 10 |  | Wrong outcome |
| Digital dietitian-pilot study of a novel smartphone application | Farfan, C. C.; Axelrod, C.; Frase, D. L.; Mackenzie, A.; Haroush, G.; Castorino, K. | 2018 | Diabetes | 67 |  | A200 | Wrong outcome |
| Dietary self-monitoring using an app: Are frequency, consistency and completeness related to weight loss? | Payne, J. E.; Turk, M. T.; Pellegrini, C. A.; Kalarchian, M. A. | 2020 | Circulation | 141 |  |  | Wrong outcome |
| Dietary self-monitoring through calorie tracking but not through a digital photography app is associated with significant weight loss: the 2SMART pilot study - a 6-month randomized trial | Dunn, C. G.; Turner-McGrievy, G. M.; Wilcox, S.; Hutto, B. | 2019 | Journal of the Academy of Nutrition and Dietetics | 119 | 9 | 1525-1532 | Wrong outcome |
| Cooking Matters Mobile Application: a meal planning and preparation tool for low-income parents | Garvin, T. M.; Chiappone, A.; Boyd, L.; Stern, K.; Panichelli, J.; Edwards Hall, L. A.; Yaroch, A. L. | 2019 | Public health nutrition | 22 | 12 | 2220-2227 | Wrong outcome |
| Being my own companion in times of social isolation- A 14-day mobile self-compassion intervention improves stress levels and eating behavior | Schnepper, Rebekka; Reichenberger, Julia; Blechert, Jens | 2020 | Frontiers in Psychology | 11 |  |  | Wrong outcome |
| Adherence to mobile-app-based dietary self-monitoring-Impact on weight loss in adults | Payne, J. E.; Turk, M. T.; Kalarchian, M. A.; Pellegrini, C. A. | 2022 | OBESITY SCIENCE & PRACTICE | 8 | 3 | 279-288 | Wrong outcome |
| Addiction model intervention for obesity: A smartphone app pilot study in obese youth | Pretlow, R.; Stock, C. | 2014 | Obesity Reviews | 15 |  | 152 | Wrong outcome |
| A phenomenological exploration of change towards healthier food purchasing behaviour in women from a lower socioeconomic background using a health app | Flaherty, S. J.; McCarthy, M. B.; Collins, A. M.; McCafferty, C.; McAuliffe, F. M. | 2020 | Appetite | 147 |  | 104566 | Wrong outcome |
| The Effect of Mindful Eating on Calorie Intake and Diet | NCT03601650 | 2018 | https://clinicaltrials.gov/show/NCT03601650 | | | | Wrong outcome |
| Development and Evaluation of a Nutritional Smartphone Application for Making Smart and Healthy Choices in Grocery Shopping | | 2017 | Healthcare informatics research | 23 | 1 | 16-24 | Wrong outcome |
| Study of an International Commercial Program on Weight Loss and Health Outcomes | NCT03571893 | 2018 | https://clinicaltrials.gov/show/NCT03571893 | | | | Wrong outcome |
| Precision Public Health: enhancing Connections to Develop Just-in-Time Adaptive Intervention Strategies | NCT03836391 | 2019 | https://clinicaltrials.gov/show/NCT03836391 | | | | Wrong outcome |
| Telehealth Program in Usual Care Setting | NCT04201028 | 2019 | https://clinicaltrials.gov/show/NCT04201028 | | | | Wrong outcome |
| The Move, Eat & Sleep study: an app-based physical activity, diet and sleep health weight loss program | ACTRN12617000735358 | 2017 | https://trialsearch.who.int/Trial2.aspx?TrialID=ACTRN12617000735358 | | | | Wrong outcome |
| Feasibility Study of Receiving Feedback Messages Based on Self-monitored Dietary Intake | NCT02829632 | 2015 | https://clinicaltrials.gov/show/NCT02829632 | | | | Wrong outcome |
| Slip Buddy App for Weight Management: randomized Feasibility Trial of a Dietary Lapse Tracking App | Pagoto, S; Tulu, B; Waring, ME; Goetz, J; Bibeau, J; Divito, J; Groshon, L; Schroeder, M | 2021 | JMIR mHealth and uHealth | 9 | 4 | e24249 | Wrong outcome |
| Using augmented reality to inform consumer choice and lower carbon footprints | Isley, S. C.; Ketcham, R.; Arent, D. J. | 2017 | Environmental Research Letters | 12 | 6 | 64002 | Wrong outcome |
| Using a Smartphone Application to Promote Healthy Dietary Behaviours and Local Food Consumption | Gilliland, Jason; Sadler, Richard; Clark, Andrew; O‚ÄôConnor, Colleen; Milczarek, Malgorzata; Doherty, Sean; O'Connor, Colleen | 2015 | BioMed Research International | 2015 |  | 01-Nov | Wrong outcome |
| The Outcomes of App-Based Health Coaching to Improve Dietary Behavior Among Nurses in a Tertiary Hospital: Pilot Intervention Study | Lim, Wei Xiang; Fook-Chong, Stephanie; Lim, John Wah; Gan, Wee Hoe | 2022 | JMIR nursing | 5 | 1 | e36811 | Wrong outcome |
| The Impact of Smartphone Apps Designed to Reduce Food Waste on Improving Healthy Eating, Financial Expenses and Personal Food Waste: Crossover Pilot Intervention Trial Studying Students' User Experiences | Mathisen, Therese Fostervold; Johansen, Frode Ramstad | 2022 | JMIR formative research | 6 | 9 | e38520 | Wrong outcome |
| The HealtheSteps TM lifestyle prescription program to improve physical activity and modifiable risk factors for chronic disease: a pragmatic randomized controlled trial | Gill, D. P.; Blunt, W.; Boa Sorte Silva, N. C.; Stiller-Moldovan, C.; Zou, G. Y.; Petrella, R. J. | 2019 | BMC public health | 19 | 1 | 841 | Wrong outcome |
| The effectiveness of a motivational enhancement smartphone application promoting lifestyle improvement for brain health: a randomized controlled trial | Roh, HyunWoong; Ryu, Hankyel; Jeong, Sooin; Han, Jieun; Park, Bumhee; Moon, SoYoung; Choi, SeongHey; Son, SangJoon; Hong, ChangHyung | 2022 | PLoS ONE | 17 | 6 |  | Wrong outcome |
| The effectiveness of a monetary reimbursement model for weight reduction via a smartphone application: a preliminary retrospective study | Lee, J.; Bae, S.; Park, D.; Kim, Y.; Park, J. | 2020 | Scientific reports | 10 | 1 | 15714 | Wrong outcome |
| The Effect of a Future-Self Avatar Mobile Health Intervention (FutureMe) on Physical Activity and Food Purchases: Randomized Controlled Trial | Monninghoff, A.; Fuchs, K.; Wu, J.; Albert, J.; Mayer, S. | 2022 | Journal of Medical Internet Research | 24 | 7 | e32487 | Wrong outcome |
| Smartphone-based cognitive bias modification training improves healthy food choice in obesity: a pilot study | Kakoschke, N.; Hawker, C.; Castine, B.; Courten, B. de; Verdejo-Garcia, A. | 2018 | European Eating Disorders Review | 26 | 5 | 526-532 | Wrong outcome |
| Smartphone technology facilitates dietary change in healthy adults | Ipjian, M. L.; Johnston, C. S. | 2017 | Nutrition | 33 |  | 343-347 | Wrong outcome |
| Short-Term Effectiveness of a Mobile Phone App for Increasing Physical Activity and Adherence to the Mediterranean Diet in Primary Care: A Randomized Controlled Trial (EVIDENT II Study) | Recio-Rodriguez, Jose I.; Agudo-Conde, Cristina; Martin-Cantera, Carlos; Gonzalez-Viejo, M. Natividad; Fernandez-Alonso, M. Del Carmen; Arietaleanizbeaskoa, Maria Soledad; Schmolling-Guinovart, Yolanda; Maderuelo-Fernandez, Jose-Angel; Rodriguez-Sanchez, Emiliano; Gomez-Marcos, Manuel A.; Garcia-Ortiz, Luis; Investigators, Evident | 2016 | Journal of medical Internet research | 18 | 12 | e331 | Wrong outcome |
| Quasi-experimental design for using an interactive social media intervention program to improve truck drivers' health beliefs and eating behaviors | Chang, SsuLan; Wu, WenChi; Hu, YihJin; Lai, HsinYi; Wong, TeChih | 2022 | BMC Public Health | 22 | 1486 |  | Wrong outcome |
| Mothers' DASH diet adherence and food purchases after week-long episodic future thinking intervention | Hollis-Hansen, K.; Seidman, J.; O'Donnell, S.; Epstein, L. H. | 2020 | Appetite |  |  | 104757 | Wrong outcome |
| Mobile apps as a sustainable shopping guide: The effect of eco-score rankings on sustainable food choice | Weber, A. | 2021 | Appetite | 167 |  | 105616 | Wrong outcome |
| Mobile app increases vegetable-based preparations by low-income household cooks: a randomized controlled trial | Clarke, P.; Evans, S. H.; Neffa-Creech, D. | 2019 | Public Health Nutrition | 22 | 4 | 714-725 | Wrong outcome |
| Machine Learning Analysis to Identify Digital Behavioral Phenotypes for Engagement and Health Outcome Efficacy of an mHealth Intervention for Obesity: Randomized Controlled Trial | Kim, Meelim; Yang, Jaeyeong; Ahn, Woo-Young; Choi, Hyung Jin | 2021 | Journal of Medical Internet Research | 23 | 6 | N.PAG-N.PAG | Wrong outcome |
| Long-Term Effectiveness of a Smartphone App for Improving Healthy Lifestyles in General Population in Primary Care: Randomized Controlled Trial (Evident II Study) | Garcia-Ortiz, Luis; Recio-Rodriguez, Jose Ignacio; Agudo-Conde, Cristina; Patino-Alonso, Maria Carmen; Maderuelo-Fernandez, Jose-Angel; Repiso Gento, Irene; Puigdomenech Puig, Elisa; Gonzalez-Viejo, Natividad; Arietaleanizbeaskoa, Maria Soledad; Schmolling-Guinovart, Yolanda; Gomez-Marcos, Manuel Angel; Rodriguez-Sanchez, Emiliano; Group, Evident Investigators; Mobilizing Minds Research, Group | 2018 | JMIR mHealth and uHealth | 6 | 4 | e107 | Wrong outcome |
| Long-term Effectiveness of a Smartphone App Combined With a Smart Band on Weight Loss, Physical Activity, and Caloric Intake in a Population With Overweight and Obesity (Evident 3 Study): Randomized Controlled Trial | Lugones-Sanchez, Cristina; Recio-Rodriguez, Jose I.; Agudo-Conde, Cristina; Repiso-Gento, Irene; Adalia, Esther G.; Ramirez-Manent, Jos√© Ignacio; Sanchez-Calavera, Maria Antonia; Rodriguez-Sanchez, Emiliano; Gomez-Marcos, Manuel A.; Garcia-Ortiz, Luis; Investigators, Evident; G Adalia, Esther | 2022 | Journal of Medical Internet Research | 24 | 2 | N.PAG-N.PAG | Wrong outcome |
| Keyto App and Device versus WW App on Weight Loss and Metabolic Risk in Adults with Overweight or Obesity: A Randomized Trial | Falkenhain, K.; Locke, S. R.; Lowe, D. A.; Reitsma, N. J.; Lee, T.; Singer, J.; Weiss, E. J.; Little, J. P. | 2021 | Obesity (Silver Spring, Md.) | | |  | Wrong outcome |
| Iterative design and testing of a mobile application to support food consumption monitoring and decision making | James, Melva Tonisha | 2016 | Dissertation Abstracts International: Section B: The Sciences and Engineering | 77 | 5 | No-Specified | Wrong outcome |
| Improvements in Diet and Physical Activity-Related Psychosocial Factors Among African Americans Using a Mobile Health Lifestyle Intervention to Promote Cardiovascular Health: The FAITH! (Fostering African American Improvement in Total Health) App Pilot St | Cyriac, J.; Jenkins, S.; Patten, C. A.; Hayes, S. N.; Jones, C.; Cooper, L. A.; Brewer, L. C. | 2021 | JMIR mHealth and uHealth | 9 | 11 | e28024 | Wrong outcome |
| Impact of the New Zealand health star rating system: RCT results and evaluation of roll-out | Ni Mhurchu, C.; Eyles, H.; Volkova, E.; Jiang, Y.; Neal, B.; Blakely, T.; Swinburn, B.; Rayner, M.; Choi, Y. H. | 2017 | Annals of Nutrition and Metabolism | 71 |  | 101 | Wrong outcome |
| Evaluation of an Application for Mobile Telephones (e-12HR) to Increase Adherence to the Mediterranean Diet in University Students: A Controlled, Randomized and Multicentric Study | Bejar, L. M.; Garcia-Perea, M. D.; Mesa-Rodriguez, P. | 2022 | Nutrients | 14 | 19 | 4196 | Wrong outcome |
| Effects of interpretive nutrition labels on consumer food purchases: the Starlight randomized controlled trial | Ni Mhurchu, Cliona; Volkova, Ekaterina; Jiang, Yannan; Eyles, Helen; Michie, Jo; Neal, Bruce; Blakely, Tony; Swinburn, Boyd; Rayner, Mike | 2017 | The American journal of clinical nutrition | 105 | 3 | 695-704 | Wrong outcome |
| Effects of digital just-in-time nudges on healthy food choice - a field experiment | Laan, L. N. van der; Orcholska, O. | 2022 | Food Quality and Preference | 98 |  |  | Wrong outcome |
| Effects of Different Types of Front-of-Pack Labelling Information on the Healthiness of Food Purchases-A Randomised Controlled Trial | Neal, Bruce; Crino, Michelle; Dunford, Elizabeth; Gao, Annie; Greenland, Rohan; Li, Nicole; Ngai, Judith; Ni Mhurchu, Cliona; Pettigrew, Simone; Sacks, Gary; Webster, Jacqui; Wu, Jason H. Y. | 2017 | Nutrients | 9 | 12 |  | Wrong outcome |
| Effects of a Smartphone-Based Approach-Avoidance Intervention on Chocolate Craving and Consumption: Randomized Controlled Trial | Meule, A.; Richard, A.; Dinic, R.; Blechert, J. | 2019 | JMIR mHealth and uHealth | 7 | 11 | e12298 | Wrong outcome |
| Effects and challenges of using a nutrition assistance system: results of a long-term mixed-method study | Hauptmann, H.; Leipold, N.; Madenach, M.; Wintergerst, M.; Lurz, M.; Groh, G.; Bohm, M.; Gedrich, K.; Krcmar, H. | 2022 | USER MODELING AND USER-ADAPTED INTERACTION | 32 | 5 | 923-975 | Wrong outcome |
| Effect Of An Mobile Health Weight Loss Intervention On Healthy Eating Index Diet Quality: The Smarter Randomized Controlled Trial | Cheng, J.; Sereika, S. M.; Costacou, T.; Conroy, M. B.; Parmanto, B.; Rockette-Wagner, B.; Kriska, A.; Burke, L. E. | 2022 | CIRCULATION | 145 |  |  | Wrong outcome |
| Do nutrition labels influence healthier food choices? Analysis of label viewing behaviour and subsequent food purchases in a labelling intervention trial | Ni Mhurchu, C.; Eyles, H.; Jiang, Y.; Blakely, T. | 2018 | Appetite | 121 |  | 360-365 | Wrong outcome |
| Digitally Supported Lifestyle Intervention to Prevent Type 2 Diabetes Through Healthy Habits: Secondary Analysis of Long-Term User Engagement Trajectories in a Randomized Controlled Trial | Lavikainen, P.; Mattila, E.; Absetz, P.; Harjumaa, M.; Lindstrom, J.; Jarvela-Reijonen, E.; Aittola, K.; Mannikko, R.; Tilles-Tirkkonen, T.; Lintu, N.; Lakka, T.; van Gils, M.; Pihlajamaki, J.; Martikainen, J. | 2022 | Journal of Medical Internet Research | 24 | 2 | e31530 | Wrong outcome |
| Dietary self-monitoring, but not dietary quality, improves with use of smartphone app technology in an 8-week weight loss trial | Wharton, Christopher M.; Johnston, Carol S.; Cunningham, Barbara K.; Sterner, Danielle | 2014 | Journal of Nutrition Education and Behavior | 46 | 5 | 440-444 | Wrong outcome |
| Chronic disease risks and use of a smartphone application during a physical activity and dietary intervention in Australian truck drivers | Gilson, N. D.; Pavey, T. G.; Vandelanotte, C.; Duncan, M. J.; Gomersall, S. R.; Trost, S. G.; Brown, W. J. | 2016 | Australian and New Zealand Journal of Public Health | 40 | 1 | 91-93 | Wrong outcome |
| Changes in lifestyles, cognitive impairment, quality of life and activity day living after combined use of smartphone and smartband technology: a randomized clinical trial (EVIDENT-Age study) | Recio-Rodr√≠guez, Jos√© I.; Gonzalez-Sanchez, Susana; Tamayo-Morales, Olaya; G√≥mez-Marcos, Manuel A.; Garcia-Ortiz, Luis; Ni√±o-Mart√≠n, Virtudes; Lugones-Sanchez, Cristina; Rodriguez-Sanchez, Emiliano | 2022 | BMC Geriatrics | 22 | 1 | 01-Dec | Wrong outcome |
| Boosting healthy food choices by meal colour variety: results from two experiments and a just-in-time Ecological Momentary Intervention | Konig, L. M.; Renner, B. | 2019 | BMC Public Health | 19 | 975 |  | Wrong outcome |
| Automated personalized feedback for physical activity and dietary behavior change with mobile phones: a randomized controlled trial on adults | Rabbi, Mashfiqui; Pfammatter, Angela; Zhang, Mi; Spring, Bonnie; Choudhury, Tanzeem | 2015 | JMIR mHealth and uHealth | 3 | 2 | e42 | Wrong outcome |
| Adherence to a caloric budget and body weight change vary by season, gender, and BMI: An observational study of daily users of a mobile health app | Labonte, K.; Knauper, B.; Dube, L.; Yang, N.; Nielsen, D. E. | 2022 | Obesity Science and Practice | 8 | 6 | 735-747 | Wrong outcome |
| A smartphone based attentive eating intervention for energy intake and weight loss: results from a randomised controlled trial | Whitelock, V.; Kersbergen, I.; Higgs, S.; Aveyard, P.; Halford, J. C. G.; Robinson, E. | 2019 | BMC Public Health | 19 | 611 |  | Wrong outcome |
| A Serious Game to Increase Healthy Food Consumption in Overweight or Obese Adults: Randomized Controlled Trial | Blackburne, Tegan; Rodriguez, Alexandra; Johnstone, Stuart John | 2016 | JMIR serious games | 4 | 2 | e10 | Wrong outcome |
| A MICROBIOTA TARGETED, MEDITERRANEAN DIET-BASED NUTRITIONAL EDUCATION PROGRAM POSITIVELY MODIFIES THE INTESTINAL ECOSYSTEM OF HEALTHY INDIVIDUALS | Godny, L.; Reshef, L.; Fischler, T. S.; Elial-Fatal, S.; Pfeffer-Gik, T.; Raykhel, B.; Rabinowitz, K. M.; Levy-Barda, A.; Perets, T. T.; Barken, R.; Goren, I.; Ollech, J.; Yanai, H. A.; Gophna, U.; Dotan, I. | 2022 | Gastroenterology | 162 | 7 | S-828 | Wrong outcome |
| A Fully Automated Conversational Artificial Intelligence for Weight Loss: Longitudinal Observational Study Among Overweight and Obese Adults | Stein, Natalie; Brooks, Kevin | 2017 | JMIR diabetes | 2 | 2 | e28 | Wrong outcome |
| A cluster randomized controlled trial feasibility study of a whatsapp-delivered intervention to promote healthy eating habits in male firefighters | Ng, W. W. M.; Wong, A. S. W.; Cheung, K. | 2021 | International Journal of Environmental Research and Public Health | 18 | 12 | 6633 | Wrong outcome |
| Evaluating Consumer m‚ÄêHealth Services for User Engagement and Health Promotion: an Organizational Field Experiment | NCT02206893 | 2014 | https://clinicaltrials.gov/show/NCT02206893 | | | | Wrong outcome |
| ImpulsePal: a feasibility study of to aid the planning of a randomised controlled trial and refinement of a smartphone app-based intervention to support weight loss | ISRCTN14886370 | 2018 | https://trialsearch.who.int/Trial2.aspx?TrialID=ISRCTN14886370 | | | | Wrong outcome |
| Social networks for improving healthy weight loss behaviors for overweight and obese adults: a randomized clinical trial of the social pounds off digitally (Social POD) mobile app | Hales, S; Turner-McGrievy, GM; Wilcox, S; Fahim, A; Davis, RE; Huhns, M; Valafar, H | 2016 | International journal of medical informatics | 94 |  | 81-90 | Wrong outcome  |
| Feasibility, acceptability, and preliminary impact of "Supper Heroes", a family-based sustainable diet intervention. | Eckert, Katherine F and Agostinelli, Julia and Laila, Amar and Alexander, Chloe and Parizeau, Kate and von Massow, Mike and Duncan, Alison M and Hesketh, Kylie D and Ma, David W L and Haines, Jess | 2025 | Appetite | 206 | 6 | 107849 | Wrong outcome |
| Personal Goals, User Engagement, and Meal Adherence within a Personalised AI-Based Mobile Application for Nutrition and Physical Activity. | Patra, Elena and Kokkinopoulou, Anna and Wilson-Barnes, Saskia and Hart, Kathryn and Gymnopoulos, Lazaros P and Tsatsou, Dorothea and Solachidis, Vassilios and Dimitropoulos, Kosmas and Rouskas, Konstantinos and Argiriou, Anagnostis and Lalama, Elena and Csanalosi, Marta and Pfeiffer, Andreas F H and Cornelissen, Veronique and Decorte, Elise and Dias, Sofia Balula and Oikonomidis, Yannis and Maria Botana, Jose and Leoni, Riccardo and Russell, Duncan and Mantovani, Eugenio and Aleksic, Milena and Brkic, Boris and Hassapidou, Maria and Pagkalos, Ioannis | 2024 | Life (Basel, Switzerland) | 14 | 10 |  | Wrong outcome |
| Short-Term Effect of a Health Promotion Intervention Based on the Electronic 12-Hour Dietary Recall (e-12HR) Smartphone App on Adherence to the Mediterranean Diet Among Spanish Primary Care Professionals: Randomized Controlled Clinical Trial. | Bejar, Luis Maria and Mesa-Rodriguez, Pedro and Garcia-Perea, Maria Dolores | 2024 | JMIR mHealth and uHealth | 12 | 101624439 | e49302 | Wrong outcome |
| How a food scanner app influences healthy food choice. | Werle, Carolina O C and Gauthier, Caroline and Yamim, Amanda P and Bally, Frederic | 2024 | Appetite | 200 | 6 | 107571 | Wrong outcome |
| A Randomized Controlled Trial, Non-Nutrition Based mHealth Program: The Potential Impact on Dietary Intake in College Students. | Tran, Dieu-My T and Cross, Chad L and Navalta, James W | 2024 | Clinical nursing research | 33 | 1 | 34-39 | Wrong outcome |
| Effectiveness of a Smartphone App (e-12HR) in Improving Adherence to the Mediterranean Diet in Spanish University Students by Age, Gender, Field of Study, and Body Mass Index: A Randomized Controlled Trial. | Bejar, Luis M and Mesa-Rodriguez, Pedro and Quintero-Florez, Angelica and Ramirez-Alvarado, Maria Del Mar and Garcia-Perea, Maria Dolores | 2023 | Nutrients | 15 | 7 |  | Wrong outcome |
| The Impact of Smartphone Apps Designed to Reduce Food Waste on Improving Healthy Eating, Financial Expenses and Personal Food Waste: Crossover Pilot Intervention Trial Studying Students' User Experiences | Mathisen, TF and Johansen, FR | 2022 | JMIR FORMATIVE RESEARCH | 6 | 9 |  | Wrong outcome |
| Multimodality dietary intervention for colorectal cancer prevention: the MyBestGI randomized trial | Djuric, Z and Segar, M and Sen, A and Kadri, R and Adwere-Boamah, R and Orr, J and Poore, K and Rifkin, S and Buis, L | 2024 |  | 84 | 7 |  | Wrong outcome |
| A Serious Game to Increase Healthy Food Consumption in Overweight or Obese Adults: Randomized Controlled Trial | Blackburne, T and Rodriguez, A and Johnstone, SJ | 2016 |  | 4 | 2 | e10 | Wrong outcome |
| Automated personalized feedback for physical activity and dietary behavior change with mobile phones: a randomized controlled trial on adults | Rabbi, M and Pfammatter, A and Zhang, M and Spring, B and Choudhury, T | 2015 |  | 3 | 2 | e42 | Wrong outcome |
| Tailored Messaging to Reduce Sodium Intake | NCT03099343 | 2017 | https://clinicaltrials.gov/show/NCT03099343 | | | | Wrong population |
| Salt ALTernatives Study (SALTS): a smartphone app and dietary alternative salt to lower blood pressure for adults with high blood pressure | ACTRN12619000352101 | 2019 | https://trialsearch.who.int/Trial2.aspx?TrialID=ACTRN12619000352101 | | | | Wrong population |
| Use of a mobile health application in wellness: An assessment of needs, perceptions, usability and efficacy in changing dietary choices in a university student population | Slazus, C.; Ebrahim, Z.; Koen, N. | 2021 | South African Journal of Clinical Nutrition | 34 | 3 | 193 | Wrong situation |
| Promoting healthy lifestyle in Chinese college students: evaluation of a social media-based intervention applying the RE-AIM framework | Wang, Mengying; Guo, Yijing; Zhang, Yu; Xie, Sasa; Yu, ZhiYing; Luo, Jun; Zhang, Danyu; Ming, Zhaoyan; Li, Xiuyang; Yang, Min | 2021 | European journal of clinical nutrition | 75 | 2 | 335-344 | Wrong situation |
| Calorie counting smart phone apps: Effectiveness in nutritional awareness, lifestyle modification and weight management among young Indian adults | Banerjee, Paromita; Mendu, Vishnu Vardhana Rao; Korrapati, Damayanthi; Gavaravarapu, SubbaRao M. | 2020 | Health Informatics Journal | 26 | 2 | 816-828 | Wrong situation |
| A digital health weight-loss intervention in severe obesity | Senecal, Conor; Collazo-Clavell, Maria; Larrabee, Beth R.; de Andrade, Mariza; Lin, Weihua; Chen, Bing; Lerman, Lilach O.; Lerman, Amir; Lopez-Jimenez, Francisco | 2020 | Digital health | 6 |  | 2.05521E+15 | Wrong situation |
| Your digital nutritionist | Hamideh, D.; Arellano, B.; Topol, E. J.; Steinhubl, S. R. | 2019 | The Lancet | 393 | 10166 | 19 | Wrong study design |
| Will Your Next Prescription be for the Pharmacy or the Farmacy? | Evans, Joanne | 2022 | New Jersey Nurse | 52 | 1 | 15-15 | Wrong study design |
| Translating health promotion research into community practice: The ManUp physical activity and nutrition project | Kolt, G.; Caperchione, C.; Duncan, M.; Vandelanotte, C.; Rosenkranz, R.; Maeder, A.; Karunanithi, M.; Mummery, K. | 2013 | Journal of Science and Medicine in Sport | 16 |  | e47-e48 | Wrong study design |
| The impact of a supermarket-based intervention using personalised loyalty card incentives to increase weekly purchasing of fruits and vegetables | Stewart, E.; Jenneson, V.; Tempest, B.; Walker, T.; Evans, C. | 2022 | Proceedings of the Nutrition Society | 81 |  | E183 | Wrong study design |
| Pilot study: Use of a novel portion control device and dietetic app in a six-week weight management intervention | McLoughlin, R.; Byrne, D. G.; McCartney, D. | 2022 | Proceedings of the Nutrition Society | 81 |  | E89 | Wrong study design |
| MyBehavior: Automatic Personalized Health Feedback from User Behaviors and Preferences using Smartphones | Rabbi, M.; Aung, M. H.; Zhang, M.; Choudhury, T.; Acm | 2015 |  |  |  | 707-718 | Wrong study design |
| Development of a clinical tool for weight management using response inhibition training | Cox, J. S.; Khalil, N. H.; Hinton, E. C.; Hamilton-Shield, J. P.; Brunstrom, J. M.; Lawrence, N. S. | 2018 | Appetite | 130 |  | 302 | Wrong study design |
| Comment on "Technology as a Tool to Encourage Young Adults to Sleep and Eat Healthy"...Adams S, Liguori G, Lofgren I. Technology as a tool to encourage young adults to sleep and eat healthy. ACSMs Health Fit J. 2017;21(4):4-6 | Javad Mortazavi, Seyed Mohammad | 2017 | ACSM's Health & Fitness Journal | 21 | 6 | 48-48 | Wrong study design |
| Choosing between responsive-design websites versus mobile apps for your mobile behavioral intervention: Presenting four case studies | Turner-McGrievy, Gabrielle M.; Hales, Sarah B.; Schoffman, Danielle E.; Valafar, Homay; Brazendale, Keith; Weaver, R. Glenn; Beets, Michael W.; Wirth, Michael D.; Shivappa, Nitin; Mandes, Trisha; Hebert, James R.; Wilcox, Sara; Hester, Andrew; McGrievy, Matthew J. | 2017 | Translational Behavioral Medicine | 7 | 2 | 224-232 | Wrong study design |
| A novel mobile app's reliability, end user satisfaction, and changes in dash diet eating patterns over 8 weeks | Steigerwalt, S.; Hummel, S.; DiFilippo, K.; Scisney-Matlock, M. | 2019 | Hypertension | 74 |  |  | Wrong study design |
| SmartEater: smartphone based interventions in everyday life | DRKS00017493 | 2019 | https://trialsearch.who.int/Trial2.aspx?TrialID=DRKS00017493 | | | | Wrong study design |
| A smart phone application for increasing fruit and vegetable knowledge and intakes: development and inital testing | Appleton, K; Passmore, D; Burn, I; Pidgeon, H; Nation, P; Boobyer, C; Jiang, N | 2020 | Proceedings of the Nutrition Society | 79 |  |  | Wrong study design |
| User Experiences of a Smartphone-Based Attentive Eating App and Their Association With Diet and Weight Loss Outcomes: thematic and Exploratory Analyses From a Randomized Controlled Trial | Whitelock, V; Kersbergen, I; Higgs, S; Aveyard, P; Halford, JC; Robinson, E | 2020 | JMIR mHealth and uHealth | 8 | 10 | e16780 | Wrong study design |
| A retrospective real-world observational pilot analysis of Waya: A self-monitoring fitness app in Germany | Balakrishnan, P.; Owen, E.; Eberl, M.; Friedrich, B.; Etter, T. | 2022 | Cardiovascular Endocrinology and Metabolism | 11 | 3 | e0266 | Wrong study design |

## **Table 10. Summary of study characteristics.**

| **Study** | **Country** | **Study design** | **Baseline (follow-up) sample size** | **Retention rate (%)** | **Intervention duration (weeks)** | **Intervention condition** | **Control condition** | **Outcome(s)** | **Longest follow-up (weeks)** |
| --- | --- | --- | --- | --- | --- | --- | --- | --- | --- |
| Appleton et al., 2019 | UK | RCT waitlist | 94 (94) | 100 | 2 | The SMART-5-A-DAY app allowed users to input and view their daily fruit and vegetable intake compared with the UK 5-a-day recommendation. The user's total fruit and vegetables inputted for the current day is displayed alongside a motivational or congratulatory message. | Waitlist | Fruit and vegetables | 14 |
| Aulbach et al., 2021 | UK | Pre-post trial | 1234 (1186) | 96.1 | 2 | The FoodT app contained daily 10-minute cognitive training sessions, consisting of a go/no-go task in which energy-dense foods were paired with a no-go signal and healthy foods with a go signal. Users could also personalise the app by selecting up to three of the following foods: alcohol, biscuits, bread, cake, cheese, chocolate, crisps, fast food, fizzy drinks, ice cream, meat, pastries, pizza, and sweets. | Baseline | Fruit and vegetables, dairy, meat | 27 |
| Bhurosy et al., 2020 | USA | RCT parallel group | 281 (165) | 58.7 | 0.43 | The app (unnamed) asked participants to count the frequency of their red/orange vegetable consumption and set a goal to eat one more portion the following day. They could use their smartphones to take photographs of their meals to remember what they ate. On the final day, participants provided photographs and descriptions of their meals and completed the counting part only. | No intervention | Fruit and vegetables | 2 |
| Brewer et al., 2019 | USA | Pre-post trial | 50 (45) | 90 | 10 | The FAITH! app involved core multimedia education modules with videos from health professionals on cardiovascular health, diet, and physical activity, self-monitoring, and social networking through a discussion board. | Baseline | Fruit and vegetables | 196 |
| Carfora and Catellani, 2022 | Italy | RCT parallel group | 425 (221) | 52 | 2 | The PsyMe app was used to deliver four interventions. Every day for two weeks, participants received persuasive messages via the app:   1. Addition condition: messages included "Legume cultivation (e.g., beans, chickpeas, lentils, peas) increases nitrogen quantity in fields, which enhances soil fertility. If you eat legumes, you protect soil fertility". 2. Addition + dynamic norm condition: messages included “Nowadays, the number of people who eat legumes (e.g., beans, chickpeas, lentils, peas) is increasing. The limited use of fertilizers for legume cultivation reduces greenhouse gas emissions. If you also eat legumes, you reduce greenhouse gas emissions". 3. Replacement condition: messages included "Legume cultivation (e.g., beans, chickpeas, lentils, peas) increases nitrogen quantity in fields, which enhances soil fertility. Cultivation for livestock feeding does not have this property. If you eat legumes instead of meat, you protect soil fertility". 4. Replacement + dynamic norm condition: messages included "Nowadays, more and more people eat legumes (e.g., beans, chickpeas, lentils, peas), instead of meat. Legume cultivation increases nitrogen quantity in yields, which enhances soil fertility. Cultivation for livestock feeding does not have this property. If you also eat legumes instead of meat, you protect soil fertility". | No message | Legumes and meat | 28 |
| Chung et al., 2021 | China | NRCT | 305 (NR) | NR | 12 | The eDietary Portal encouraged twelve weeks of dietary monitoring. Dietary reports with nutrient analysis were shown online for the participants to review. They were asked to change their food choices day by day and eat to meet their goal. | One three-hour nutrition seminar. | Fruit and vegetables | 84 |
| Eisenhauer et al., 2021 | USA | RCT parallel group | 80 (74) | 92.5 | 24 | The LoseIt! app allowed for real-time self-monitoring of eating and activity and included personalised reports and opportunities for goal setting. There was a private discussion board, allowing sharing of personal self-monitoring strategies and experiences. Another feature was its ability to sync weight as measured by a smart scale and provide feedback on weight trends. The group also received one-way text messages containing content on healthy eating and physical activity and reminders to self-monitor. | The basic app with daily manual logging of weight, diet, and activity. | Fruit and vegetables | 84 |
| Elbert et al., 2016 | Netherlands | RCT parallel group | 228 (98) | 43 | 24 | The Groente & Fruit hAPP included functions to formulate personal action plans, carry out self-monitoring, and access fruit and vegetable recipes. Users were asked a series of questions at baseline to tailor the five messages sent out over the intervention period. Audio messages focused on the topics of wellbeing, nutrition, disease risk, and barriers to fruit and vegetable consumption. Users could go back and listen to these as they pleased. The app also sent out testimonials on success stories of people increasing their fruit and vegetable intake. | Baseline | Fruit and vegetables, legumes, meat | 168 |
| Gonzalez-Ramirez et al., 2022 | Spain | RCT parallel group | 40 (40) | 100 | 12 | The SAIBI educa app provided personalised dietary counseling based on the Mediterranean diet. The app estimated users' energy requirements based on their data and enabled users to self-monitor their daily diet by selecting foods and recipes from the app database. The app also delivered tailored feedback including recommendations on how to balance their diet, general nutrition messages, and infographics regarding basic concepts. | Standard advanced dietary counseling on healthy eating, delivered in primary healthcare centres. Counseling consisted of four weekly sessions, lasting between 60 and 90 minutes, focused on the Mediterranean diet, understanding nutrients and food labeling, and physical activity. | Fruit and vegetables | 84 |
| Hahn et al., 2021 | USA | RCT parallel group | 200 (192) | 96 | 4 | The MyFitnessPal app was set up for participants to self-monitor their diets. Daily reminders were sent at 10am for 30 days. Participants were instructed not to link MyFitnessPal to any other smartphone app. | Baseline | Fruit and vegetables | 30 |
| Hendrie et al., 2020 | Australia | Pre-post trial | 5062 (1224) | 24.2 | 3 | The VegEze app contained a 21-day challenge to encourage participants to eat different vegetables in their dinner or main meal. The app contained fun ways to help establish this habit and encouraged individuals to monitor their vegetable intake with an easy-to-use tracker for logging the amount and types of vegetables consumed at each meal. Participants could search for vegetables and record the amount they consumed in servings. | Baseline | Fruit and vegetables | 21 |
| Inauen et al., 2017 | Switzerland | RCT parallel group | 97 (70) | 72.2 | 1.43 | WhatsApp was used for a social support messaging group chat starting on day 4 for seven days. Small chat groups were assigned and moderators responded with supportive messages to any message posted. If suitable, moderators followed a list of supportive responses to common support demands, otherwise, they wrote unstandardised supportive responses. | Information on healthy eating presented by the intervention deliverers at the baseline assessment. Information was presented verbally and written on a factsheet, tailored to the participant’s randomly assigned eating goal (increasing fruit and vegetables or decreasing unhealthy snacks). | Fruit and vegetables | 7 |
| Kliemann et al., 2019 | UK | RCT parallel group | 55 (36) | 65.5 | 12 | The TopTips app was used to deliver two interventions. Both were based on habit theory to encourage a set of weight management behaviors alongside advice about repetition of behaviours:   1. The first intervention was an app that included tracking features, daily reminders, and notifications to encourage different habits. 2. The second intervention was an app that additionally included self-regulatory strategies for dealing with tempting foods. | Waitlist | Fruit and vegetables | 84 |
| Mummah et al., 2016 | USA | RCT parallel group | 17 (12) | 70.6 | 12 | Vegethon was a theory-driven app enabling self-monitoring of vegetable consumption, goal setting, feedback, and social comparison. The app also included a range of features designed to engage the user (e.g., surprise challenges, leaderboards, and weekly reports). | Waitlist | Fruit and vegetables | 56 |
| Mummah et al., 2017 | USA | RCT parallel group | 135 (126) | 93.3 | 8 | Vegethon was a theory-driven app enabling self-monitoring of vegetable consumption, goal setting, feedback, and social comparison. The app also included a range of features designed to engage the user (e.g., surprise challenges, leaderboards, and weekly reports). | Waitlist | Fruit and vegetables | 35 |
| Nezami et al., 2022 | USA | RCT parallel group | 72 (66) | 91.7 | 24 | The PATH app included dietary lessons, text messages, self-monitoring of diet, activity, and weight, and weekly personalised feedback. | Baseline | Fruit and vegetables, dairy | 168 |
| Palacios et al., 2018 | Puerto Rico | RCT parallel group | 51 (31) | 60.8 | 8 | The MyNutriCart app aimed to help participants make smart and healthy choices when purchasing foods at grocery stores. The app automatically generated healthy grocery lists following DGA recommendations and accounted for the family’s nutritional needs. The lists were personalised and unique, with the participants setting a budget and a time frame for the shop to cover, e.g., $100 for 7 days. | One face-to-face dietary counseling session. | Fruit and vegetables, legumes | 56 |
| Parr et al., 2021 | England | Pre-post trial | 68942 (8305) | 12 | 12 | The NHS Weight Loss Plan app involved a 12-week weight loss plan, originally available on the NHS website. The guides included weekly food and activity charts to record calories, exercise, and fruit and vegetable intake. They also included healthy eating, diet and exercise advice. | Baseline | Fruit and vegetables | 56 |
| Price et al., 2020 | Wales | Pre-post trial | 82 (71) | 86.6 | 8 | The app (unnamed) included a construal level mind-set task, which was linked to mealtime and cue reminders, and an optional food log. The reasons users gave for wanting to improve their eating habits were presented back to them just before they would eat in a timely fashion. Users were instructed that they could update their answers at any time if they felt that their answers have changed. There was also a just-in-time “crave buster”, which users could select at any time they were craving unhealthy food and it would present one of their healthy eating mind-set cues back to them. The food log was an optional component that users could monitor their food intake if they wished. | Baseline | Fruit and vegetables | 84 |
| Recio-Rodriguez et al., 2018 | Spain | RCT parallel group | 833 (715) | 85.8 | 12 | The EVIDENT II app involved dietary self-monitoring and tailored feedback according to adherence to dietary recommendations. The app was configured according to the participants age, sex, weight and height, and personalised recommendations were provided based on their intake. On the final day, the participants were offered a recommended plan for the following days. | Basic dietary counselling on diet and physical activity. | Fruit and vegetables, legumes, nuts, fish, dairy, meat | 336 |
| Wilson et al., 2023 | New Zealand | RCT parallel group | 186 (127) | 68.3 | 16 | The app (unnamed) comprised a personalised physical activity programme and habit-based goal setting content for healthy eating and sleep quality. The healthy eating goal was standard for all users (at least 5 servings of fruit and vegetables per day), but users were instructed to select a priority habit from 10 top tips for healthy eating that they would like to focus on. Users were then instructed to implement their chosen habit goal during the intervention, aided by completing a weekly habit checklist to support habit formation. | Waitlist | Fruit and vegetables | 16 |

## **Table 11. Summary of interventions and outcome measurements.**

| **Study** | **N time-points** | **Time-point(s) (weeks)** | **N interventions** | **N outcomes** | **N results** | **Fruit and/or vegetables** | **Legumes** | **Nuts** | **Fish** | **Dairy** | **Meat** | **Change in primary outcome** |
| --- | --- | --- | --- | --- | --- | --- | --- | --- | --- | --- | --- | --- |
| Appleton et al., 2019 | 2 | 1, 2 | 1 | 1 | 2 | 2 |  |  |  |  |  | Y |
| Aulbach et al., 2021 | 1 | 4 | 1 | 2 | 3 | 2 |  |  |  |  | 1 | N |
| Bhurosy et al., 2020 | 1 | 0.43 | 1 | 1 | 1 | 1 |  |  |  |  |  | Y |
| Brewer et al., 2019 | 1 | 28 | 1 | 1 | 1 | 1 |  |  |  |  |  | Y |
| Carfora and Catellani, 2022 | 2 | 2, 4 | 4 | 2 | 16 |  | 8 |  |  |  | 8 | Y^a^ |
| Chung et al., 2021 | 1 | 12 | 1 | 1 | 2 | 2 |  |  |  |  |  | Y |
| Eisenhauer et al., 2021 | 2 | 12, 24 | 1 | 1 | 1 | 2 |  |  |  |  |  | N |
| Elbert et al., 2016 | 1 | 24 | 1 | 1 | 1 | 1 |  |  |  |  |  | Y |
| Gonzalez-Ramirez et al., 2022 | 6 | 1, 2, 3, 4, 8, 12 | 1 | 2 | 12 | 6 | 3 |  |  |  | 3 | Y |
| Hahn et al., 2021 | 1 | 4 | 1 | 3 | 3 | 3 |  |  |  |  |  | N |
| Hendrie et al., 2020 | 1 | 3 | 1 | 1 | 1 | 1 |  |  |  |  |  | Y |
| Inauen et al., 2017 | 1 | 1.86 | 1 | 1 | 1 | 1 |  |  |  |  |  | N |
| Kliemann et al., 2019 | 1 | 12 | 2 | 1 | 2 | 2 |  |  |  |  |  | Y^b^ |
| Mummah et al., 2016 | 1 | 12 | 1 | 1 | 1 | 1 |  |  |  |  |  | Y |
| Mummah et al., 2017 | 1 | 5 | 1 | 1 | 1 | 1 |  |  |  |  |  | N |
| Nezami et al., 2022 | 1 | 24 | 1 | 2 | 3 | 2 |  |  |  | 1 |  | Y |
| Palacios et al., 2018 | 1 | 8 | 1 | 4 | 7 | 2 | 1 |  |  | 2 | 2 | Y |
| Parr et al., 2021 | 1 | 12 | 1 | 1 | 1 | 1 |  |  |  |  |  | N |
| Price et al., 2020 | 1 | 8 | 1 | 1 | 2 | 2 |  |  |  |  |  | N |
| Recio-Rodriguez et al., 2018 | 2 | 12, 48 | 1 | 6 | 18 | 4 | 2 | 2 | 2 | 2 | 6 | Y |
| Wilson et al., 2023 | 1 | 16 | 1 | 1 | 1 | 1 |  |  |  |  |  | Y |

^a^Two interventions (Groups 2 and 4) were effective.

^b^One intervention (Group 2) was effective.

## **Table 12. Coding framework for combinations of behaviour change techniques and delivery techniques.**

|  | *1. Goals and planning* | *2. Feedback and monitoring* | *3. Social support* | *4. Shaping knowledge* | *5. Natural consequences* | *6. Comparison of behaviour* | *7. Associations* | *9. Comparison of outcomes* | *10. Reward and threat* | *12. Antecedents* | *13. Identity* | *15. Self-belief* |
| --- | --- | --- | --- | --- | --- | --- | --- | --- | --- | --- | --- | --- |
| Appleton et al., 2019 |  | 2 * 6 2 * 9 |  |  |  |  |  |  | 10 * 5 10 * 8 |  |  |  |
| Aulbach et al., 2021 |  |  |  |  |  |  | 7 * 7 7 * 8 7 * 9 |  |  |  |  |  |
| Bhurosy et al., 2020 | 1 * 3 | 2 * 3 2 * 5 |  |  |  |  |  |  |  |  |  |  |
| Brewer et al., 2019 |  |  | 3 * 5 |  | 5 * 8 |  |  |  |  |  |  |  |
| Carfora and Catellani, 2022a |  |  |  |  | 5 * 5 |  |  |  |  |  |  |  |
| Carfora and Catellani, 2022b |  |  |  |  | 5 * 5 | 6 * 5 |  |  |  |  |  |  |
| Carfora and Catellani, 2022c |  |  |  |  | 5 * 5 |  |  | 9 * 5 |  |  |  |  |
| Carfora and Catellani, 2022d |  |  |  |  | 5 * 5 | 6 * 5 |  | 9 * 5 |  |  |  |  |
| Chung et al., 2021 |  | 2 * 3 2 * 6 2 * 9 |  |  |  |  |  |  |  |  |  |  |
| Eisenhauer et al., 2021 |  | 2 * 5 | 3 * 5 | 4 * 5 |  |  |  |  |  |  |  |  |
| Elbert et al., 2016 | 1 * 9 | 2 * 3 |  | 4 * 8 | 5 * 5 | 6 * 5 |  |  |  |  |  |  |
| Gonzalez-Ramirez et al., 2022 |  | 2 * 6  2 * 9 |  | 4 * 1 4 * 5 4 * 8 |  |  |  |  |  |  |  |  |
| Hahn et al., 2021 |  | 2 * 2 2 * 3 |  |  |  |  |  |  |  |  |  |  |
| Hendrie et al., 2020 | 1 * 3 |  | 3 * 1 3 * 5 3 * 8 | 4 * 6 |  |  |  |  |  |  |  |  |
| Inauen et al., 2017 |  | 2 * 6 | 3 * 5 |  |  |  |  |  |  |  |  |  |
| Kliemann et al., 2019a | 1 * 2 | 2 * 3 |  | 4 * 8 4 * 9 |  |  |  |  |  |  |  |  |
| Kliemann et al., 2019b | 1 * 2 | 2 * 3 |  | 4 * 8 4 * 9 |  |  |  |  |  | 12 * 6 |  |  |
| Mummah et al., 2016 | 1 * 3 | 2 * 3  2 * 6 |  | 4 * 1 4 * 5 4 * 8 |  | 6 * 7 |  | 9 * 7 | 10 * 6 |  | 13 * 9 | 15 * 1 |
| Mummah et al., 2017 | 1 * 3 | 2 * 3  2 * 6 |  | 4 * 1 4 * 5 4 * 8 |  | 6 * 7 |  | 9 * 7 | 10 * 6 |  | 13 * 9 | 15 * 1 |
| Nezami et al., 2022 | 1 * 3 | 2 * 5 | 3 * 5 | 4 * 1 |  |  |  |  |  |  |  |  |
| Palacios et al., 2018 | 1 * 6 1 * 8 1 * 9 |  |  | 4 * 8 |  |  |  |  |  |  |  |  |
| Parr et al., 2021 |  |  |  | 4 * 1 |  |  |  |  |  |  | 13 * 3 |  |
| Price et al., 2020 | 1 * 9 | 2 * 3 |  |  | 5 * 5 |  |  |  |  |  | 13 * 9 |  |
| Recio-Rodriguez et al., 2018 | 1 * 9 1 * 6 | 2 * 3 |  |  |  |  |  |  |  |  |  |  |
| Wilson et al., 2023 | 1 * 3 | 2 * 9 |  |  |  |  |  |  |  |  |  |  |

The first numbers in the cells represent the behaviour change techniques (clusters named in row 1) and the second numbers represent the delivery techniques that are used to operationalise that BCT: 1) Information delivery, 2) Notifications, 3) Logs, 4) Passive data collection, 5) Messaging, 6) Reports, 7) Gamification, 8) Media, 9) Personalisation.

## **Table 13. Sensitivity analysis comparing the effects of specific and general apps.**

|  | **Specific apps** | | | | **General apps** | | |
| --- | --- | --- | --- | --- | --- | --- | --- |
| **Variable** | **k** | **MD** | **95% CI** |  | **k** | **MD** | **95% CI** |
| Fruit and vegetables | 5 | 0.47 | 0.36, 0.59 |  | 15 | 0.49 | 0.11, 0.86 |
| Legumes | 4 | 0.03 | 0.002, 0.07 |  | 3 | -0.01 | -0.08, 0.07 |
| Meat | 4 | -0.16 | -0.23, -0.09 |  | 4 | -0.02 | -0.05, -0.01 |

## **Table 14. Bivariate associations between exposure and outcome variables.**

|  | **Variables** | **Fruit and vegetables** | **Legumes** | **Meat** |
| --- | --- | --- | --- | --- |
| 1 | Analysis type (within vs. between) | 0.34 | 0.008 | 0.002 |
| 2 | Dietary measurement (FFQ/24h recall vs. single question/NR) | 0.13 | 0.08 | 0.04 |
| 3 | Participant type (selective vs. general population) | 0.68 | 0.38 | 0.44 |
| 4 | Sex (% female) | 0.40 | 0.08 | 0.008 |
| 5 | Age (y) | 0.27 | 0.48 | 0.53 |
| 6 | Transparency score | 0.97 | 0.02 | 0.09 |
| 7 | Baseline dietary intake | 0.11 | 0.88 | 0.02 |
| 8 | Intervention duration | 0.77 | 0.81 | 0.61 |
| 9 | Follow-up period | 0.48 | 0.94 | 0.93 |
| 10 | Goals and planning | 0.44 | 0.11 | 0.04 |
| 11 | Feedback and monitoring | 0.83 | 0.50 | 0.59 |
| 12 | Social support | 0.13 | 0.75 | 0.95 |
| 13 | Shaping knowledge | 0.68 | 0.18 | 0.22 |
| 14 | Natural consequences | 0.03 | 0.03 | 0.04 |
| 15 | Comparison of behaviour | 0.03 | 0.18 | 0.22 |
| 16 | Information delivery | 0.22 | 0.38 | 0.44 |
| 17 | Logs | 0.51 | 0.38 | 0.44 |
| 18 | Messaging | 0.70 | 0.008 | 0.01 |
| 19 | Reports | 0.90 | 0.08 | 0.10 |
| 20 | Media | 0.90 | 0.18 | 0.10 |
| 21 | Personalisation | 0.35 | 0.08 | 0.04 |

FFQ: food frequency questionnaire; NR: not reported.

Analysis type: reference group = between, 1 = within.

Dietary measurement: reference group = FFQ/24h recall, 1 = single question/NR.

Participant type: reference group = general population, 1 = selective population.

Intervention components (goals and planning, feedback and monitoring, social support, shaping knowledge, natural consequences, comparison of behaviour, information delivery, logs, messaging, reports, media, personalisation): reference group = component not included, 1 = component included.

## **Table 15. Meta-regression results for exposure and outcome variables.**

| **Fruits and vegetables** | | | |  | **Legumes** | | | |  | **Meat** | | | |
| --- | --- | --- | --- | --- | --- | --- | --- | --- | --- | --- | --- | --- | --- |
|  | Estimate | p | VIF |  |  | Estimate | p | VIF |  |  | Estimate | p | VIF |
| **Model 1** |  |  |  |  | **Model 1** |  |  |  |  | **Model 1** |  |  |  |
| Natural consequences | -0.35 | 0.42 | 1.08 |  | Analysis type | 0.05 | 0.04 | 1.75 |  | Analysis type | -0.01 | 0.96 | 42.3 |
|  |  |  |  |  | Dietary measurement | 0.13 | 0.05 | 6.89 |  | Dietary measurement | -4.61 | 0.38 | 19899 |
| Comparison of behaviour | 0.49 | 0.30 | 1.08 |  | Sex | -0.006 | 0.06 | 5.39 |  | Sex | 0.20 | 0.37 | 16919 |
|  |  |  |  |  |  |  |  |  |  | Transparency score | 0.06 | 0.43 | 906.8 |
|  |  |  |  |  |  |  |  |  |  | Baseline intake | -3.39 | 0.41 | 1715 |
|  |  |  |  |  |  |  |  |  |  |  |  |  |  |
|  |  |  |  |  |  |  |  |  |  |  |  |  |  |
|  |  |  |  |  | **Model 2** | |  |  |  | **Model 2** | | |  |
|  |  |  |  |  | Analysis type | 0.02 | 0.43 | 2.12 |  | Analysis type | -0.76 | 0.28 | 275 |
|  |  |  |  |  | Transparency | 0.01 | 0.06 | 2.18 |  | Natural consequences | 0.85 | 0.25 | 406 |
|  |  |  |  |  | Natural consequences | 0.03 | 0.35 | 3.51 |  | Messaging | -0.21 | 0.04 | 4.88 |
|  |  |  |  |  |  |  |  |  |  | Media | 0.73 | 0.30 | 627 |
|  |  |  |  |  |  |  |  |  |  |  |  |  |  |
|  |  |  |  |  |  |  |  |  |  |  |  |  |  |
|  |  |  |  |  |  | | |  |  | **Model 3** | | |  |
|  |  |  |  |  |  |  |  |  |  | Messaging | -0.24 | 0.03 | 6.42 |
|  |  |  |  |  |  |  |  |  |  | Reports | -0.03 | 0.47 | 1.33 |
|  |  |  |  |  |  |  |  |  |  | Personalisation | -0.14 | 0.14 | 6.33 |
|  |  |  |  |  |  |  |  |  |  |  |  |  |  |

## **References**

1 GOV.UK. The Eatwell Guide. 2018. <https://www.gov.uk/government/publications/the-eatwell-guide>. Accessed 22 Feb 2023.

2 U.S. Department of Agriculture and U.S. Department of Health and Human Services Dietary Guidelines for Americans, 2020-2025. 9th Edition. 2020. <https://www.dietaryguidelines.gov/resources/2020-2025-dietary-guidelines-online-materials>. Accessed 20 Jan 2025.

3 Chinese Center for Disease Control and Prevention. Eight key recommendations from Dietary Guidelines for Chinese Residents (2022). 2022. <https://en.chinacdc.cn/health_topics/nutrition_health/202206/t20220616_259702.html>. Accessed 27 Jan 2025.

4 European Commission. Food-Based Dietary Guidelines recommendations for fruit and vegetables. 2025. <https://knowledge4policy.ec.europa.eu/health-promotion-knowledge-gateway/food-based-dietary-guidelines-europe-table-3_en>. Accessed 23 Aug 2024.

5 National Health and Medical Research Council. Vegetables and legumes / beans. 2021. <https://www.eatforhealth.gov.au/food-essentials/five-food-groups/vegetables-and-legumes-beans>. Accessed 27 Jan 2025.

6 NZ Nutrition Foundation. Food Groups. 2023. <https://nutritionfoundation.org.nz/nutrition-facts/food-groups/>. Accessed 31 Jan 2025.

7 European Commission. Food-Based Dietary Guidelines recommendations for legumes. 2025. <https://knowledge4policy.ec.europa.eu/health-promotion-knowledge-gateway/food-based-dietary-guidelines-europe-table-6_en>. Accessed 27 Jan 2025.

8 European Commission. Food-Based Dietary Guidelines recommendations for meat. 2025. <https://knowledge4policy.ec.europa.eu/health-promotion-knowledge-gateway/food-based-dietary-guidelines-europe-table-8_en>. Accessed 20 Jan 2025.

9 American Heart Association. Suggested Servings From Each Food Group. 2024. <https://www.heart.org/en/healthy-living/healthy-eating/eat-smart/nutrition-basics/suggested-servings-from-each-food-group>. Accessed 23 Aug 2024.

10 Porter S, BDA. Portion sizes. 2021. <https://www.bda.uk.com/resource/food-facts-portion-sizes.html>. Accessed 23 Aug 2024.

11 Grains & Legumes Nutrition Council. Recommended Amount of Legumes. 2025. <https://www.glnc.org.au/resource/legumes-recommendations/#:~:text=As%20an%20alternative%20to%20meat%20one%20serve%20is%201%20cup,canned%20beans%20OR%20170g%20tofu>. Accessed 23 Aug 2024.

12 European Commission. Food-Based Dietary Guidelines recommendations for milk and dairy products. 2025. <https://knowledge4policy.ec.europa.eu/health-promotion-knowledge-gateway/food-based-dietary-guidelines-europe-table-7_en>. Accessed 13 Feb 2025.

13 National Health and Medical Research Council. Australian Dietary Guidelines - Providing the scientific evidence for healthier Australian Diets. Table 2.12. 2013. <https://www.nhmrc.gov.au/adg>. Accessed 13 Feb 2025.
