# Supplementary material for: The effectiveness of mobile app-based interventions in facilitating behaviour change towards healthier and more sustainable diets: a systematic review and meta-analysis
Source: Int J Behav Nutr Phys Act. 2025 Sep 30;22:122. doi: 10.1186/s12966-025-01823-7 (PMC12487266; doi:10.1186/s12966-025-01823-7)
Supplement: Supplementary file 2 — Supplementary Material 2. [file 12966_2025_1823_MOESM2_ESM.docx]

**The effectiveness of mobile app-based interventions in facilitating behaviour change towards healthier and more sustainable diets: a systematic review and meta-analysis.**

**Additional File 2**

E Curtin, R Green, KA Brown, S Nájera Espinosa, A Chandrasekar, L Hopkins, G Turner, C Alae-Carew, K Ullian, P Scheelbeek

**Table of contents**

[Figure 1. Scatter plot showing the change in food consumption across the studies. 3](#_Toc199863610)

[Figure 2. Funnel plots for publication bias of studies evaluating (from top to bottom) fruit and vegetables, legumes, dairy, and meat intake. 4](#_Toc199863611)

## **Figure 1. Scatter plot showing the change in food consumption across the studies.**

##
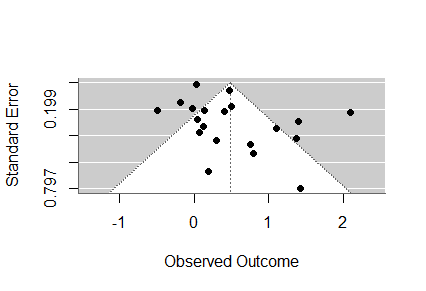


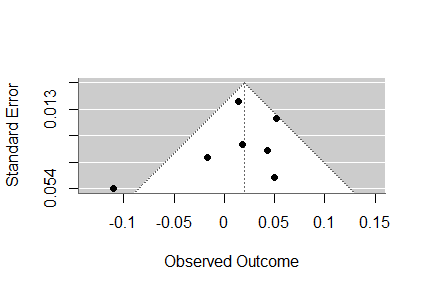


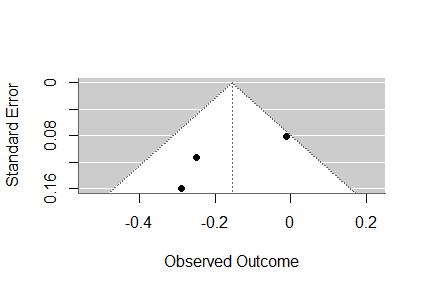


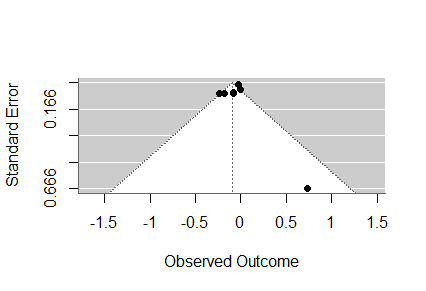


## **Figure 2. Funnel plots for publication bias of studies evaluating (from top to bottom) fruit and vegetables, legumes, dairy, and meat intake.**
